# Fludrocortisone dose–response relationship in septic shock: a randomised phase II trial

**DOI:** 10.1007/s00134-024-07616-z

**Published:** 2024-09-05

**Authors:** James Walsham, Naomi Hammond, Antje Blumenthal, Jeremy Cohen, John Myburgh, Simon Finfer, David Evans, Sandra Peake, Peter Kruger, James McCullough, Loki Johnk, Dhaval Ghelani, Laurent Billot, Sana Shan, Jason Meyer, Dorrilyn Rajbhandari, Carolyn Koch, Rinaldo Bellomo, Louise M. Burrell, Morag Young, Michael Roberts, Lorraine Mackenzie, Gregory Medley, Joshua Dalton, Balasubramanian Venkatesh

**Affiliations:** 1 Princess Alexandra Hospital, Brisbane, Australia; 2 The George Institute for Global Health, Sydney, Australia; 3 Royal North Shore Hospital, Sydney, Australia; 4 University of New South Wales, Sydney, Australia; 5 University of Queensland, Brisbane, Australia; 6 The Wesley Hospital, Brisbane, Australia; 7 Royal Brisbane Hospital, Brisbane, Australia; 8 St. George Hospital, Sydney, Australia; 9 School of Public Health, Faculty of Medicine, Imperial College London, London, UK; 10 The Queen Elizabeth Hospital, Adelaide, SA Australia; 11 University of Adelaide, Adelaide, Australia; 12 Gold Coast University Hospital, Gold Coast, QLD Australia; 13 Mater Hospital, Brisbane, Australia; 14 Blacktown Hospital, Sydney, Australia; 15 University of Sydney, Sydney, Australia; 16 Western Sydney University, Sydney, Australia; 17 Austin Hospital, Melbourne, Australia; 18 Australia and New Zealand Research Centre, Melbourne, Australia; 19 Department of Medicine, University of Melbourne, Austin Health, Australia; 20 Baker Heart and Diabetes Institute, Melbourne, Australia; 21 Therapeutics Research Centre, Basil Hetzel Institute for Translational Health Research, The Queen Elizabeth Hospital, Adelaide, Australia; 22 University of South Australia, Adelaide, Australia

**Keywords:** Fludrocortisone, Hydrocortisone, Septic shock, Pharmacokinetics

## Abstract

**Background:**

The combination of intravenous hydrocortisone and enteral fludrocortisone may reduce mortality in patients with septic shock. The optimal dose and reliability of absorption of fludrocortisone in critically ill patients are unclear.

**Methods:**

In a multi-centre, open label, phase II randomized clinical trial, intravenous hydrocortisone alone or in combination with one of three doses of enteral fludrocortisone (50 µg, 100 µg or 200 µg daily) for 7 days was compared in patients with septic shock. The primary outcome was time to shock resolution. We conducted pharmacokinetic studies to assess absorption.

**Results:**

Out of 153 enrolled patients, 38 (25%) received hydrocortisone alone, 42 (27%) received additional 50 µg, 36 (24%) received 100 µg and 37 (24%) received 200 µg fludrocortisone. Plasma concentrations of fludrocortisone were detected in 97% of patients at 3 h-median (interquartile range [IQR]) 261 (156–334) ng/L. There was no significant difference in the time to shock resolution between groups with median (IQR) of 3 (2.5–4.5), 3 (2–4), 3 (2–6) and 3 (2–5.5) days in the hydrocortisone alone, 50 µg, 100 µg and 200 µg fludrocortisone groups, respectively. The corresponding 28-day mortality rates were 9/38 (24%), 7/42 (17%), 4/36 (11%) and 4/37 (11%), respectively. There were no significant differences between groups with respect to, recurrence of shock, indices of organ failure or other secondary outcomes.

**Conclusions:**

Enteral fludrocortisone resulted in detectable plasma fludrocortisone concentrations in the majority of critically ill patients with septic shock, although they varied widely indicating differing absorption and bioavailability. Its addition to hydrocortisone was not associated with shorter time to shock resolution.

**Supplementary Information:**

The online version contains supplementary material available at 10.1007/s00134-024-07616-z.

## Take-home message

**Table:** 

This multi-centre open label randomised clinical trial in critically ill patients with septic shock provides new information on pharmacokinetics and the effects of different doses of fludrocortisone on time to shock reversal.	

## Introduction

Sepsis and septic shock are leading causes of morbidity and mortality worldwide [1]. In 2018, two large randomised clinical trials reported the effect of adjunctive corticosteroid therapy in patients with septic shock. The Adjunctive Glucocorticoid Therapy In Septic Shock Trial (ADRENAL) trial reported that a daily dose of 200 mg hydrocortisone was associated with a shorter time to resolution of shock compared to placebo but found no significant difference in 90-day mortality (the primary outcome) [2]. In contrast, the Activated Protein C and Corticosteroids for Human Septic Shock (APROCCHSS) trial reported that a combination of an intravenous dose of 200 mg hydrocortisone combined with 50 µg enteral fludrocortisone (FC) vs placebo led to a reduction in 90-day mortality [3]. Whilst hydrocortisone at a dose of 200 mg/day is recommended therapy for septic shock by the Surviving Sepsis Campaign guidelines [4] there are currently no recommendations regarding the use of fludrocortisone. An international survey demonstrated 79% of clinicians do not prescribe fludrocortisone for septic shock [5]. Uncertainty remains whether the addition of fludrocortisone to 200 mg/day hydrocortisone provides additional clinical benefit.

There are limited data on fludrocortisone pharmacokinetics. In studies in healthy volunteers, Banda et al. (*n* = 60) and Mitsky et al. (*n* = 12) reported that enterally administered fludrocortisone resulted in demonstrable plasma concentrations with terminal half-lives of 2.57 and 3.54 h, respectively [6, 7]. Vogt et al*.* reported terminal half-lives of 4.19 h and 5.52 h in 3 subjects with various disease states after intravenous and oral dosing [8]. Pharmacokinetic data on fludrocortisone in patients with septic shock are limited: in a pharmacokinetic study of enteral fludrocortisone in 21 patients with septic shock, 7 did not have detectable fludrocortisone levels; peak plasma levels were attained at 3 h and plasma half-life was 1.35 h [9]. There is limited information on adverse effects and safety profile of varying doses of fludrocortisone in the presence of concomitant hydrocortisone therapy.

We designed the Fludrocortisone Dose–Response Relationships and Vascular Responsiveness in Septic Shock (FluDReSS) study to test the hypotheses that the addition of fludrocortisone to hydrocortisone results in shorter time to resolution of shock as compared to hydrocortisone alone, and that this improvement is achieved in a dose-dependent manner. Secondary objectives of the study were to assess reliability of enteral absorption and report on any adverse effects.

## Methods

### Study design and setting

FluDReSS was an investigator-initiated, multi-centre, open-label randomised phase II clinical trial that compared intravenous hydrocortisone alone or in combination with one of three different doses of fludrocortisone in mechanically ventilated (invasive or non-invasive) patients with septic shock. The trial was conducted in nine centres in Australia.

The Trial Management Committee designed the trial that was sponsored by the George Institute for Global Health. Trained research coordinators collected the data which were entered onto a web-based database. Monitoring of the trial was conducted by The George Institute (electronic supplementary material [ESM], Table S1). The trial was registered on Clinical Trials.gov (NCT04494789).

Approval for the study was obtained from a Metro South Hospital and Health Service Human Research Ethics Committee (HREC/2019/QMS/57886) and the study has been performed in accordance with the ethical standards laid down in the 1964 Declaration of Helsinki and its later amendments. Participants or their legally authorised representative provided written informed consent or consent to continue according to the legal requirements in each jurisdiction.

### Study population and randomisation

Eligible patients were patients aged 18 and over with documented or strongly suspected infection; had at least two of the four Systemic Inflammatory Response Syndrome [10] criteria; were being treated with both ventilatory and vasopressor support and treatment with adjunctive hydrocortisone at a dose of 200 mg/day for the management of septic shock.

Patients were excluded if they were receiving long-term corticosteroids or fludrocortisone; could not receive enteric medication; death was deemed imminent or inevitable during admission or had met all inclusion criteria for more than 24 h (detailed inclusion–exclusion criteria are presented in ESM, Table S2).

### Randomisation

Eligible patients were randomised by permuted block with variable block sizes stratified by site via a password protected web-based interface (Redcap®) to either continuation of hydrocortisone alone or one of 3 different dosing regimens of additional enteral fludrocortisone.

### Trial regimen

Patients were assigned to either continue hydrocortisone alone or receive additional enteral fludrocortisone 50 µg once daily, twice daily (100 µg/day) or four times daily (200 µg/day) either orally or via a gastric tube. Detailed dosing and scheduling information is provided in ESM, Table S3. The trial regimen was continued for a maximum of seven days or until intensive care unit (ICU) discharge, cessation of hydrocortisone by the clinician or death, whichever occurred first.

### Collection of blood samples

#### Fludrocortisone assays

Blood samples for fludrocortisone levels were taken prior to and 3 h after fludrocortisone dosing. As per protocol, pharmacokinetic studies could be conducted on any day between day 1 and day 5 of enrolment. A liquid chromatography–tandem mass spectrometry (LC–MS/MS) method was developed to assay fludrocortisone levels. Details of the assay are provided in ESM, Table S4.

### Primary outcome

The primary outcome was time to resolution of shock, defined as the time from randomisation to the attainment of a clinician-prescribed mean arterial pressure target for more than 24-h without the use of vasopressors or inotropes.

### Secondary outcomes

Secondary outcomes were the recurrence of shock (defined as a new requirement for vasopressors or inotropes following resolution of shock), ventilator-free days, maximum sequential organ failure assessment (SOFA) score [11] (SOFA max), change in SOFA score (SOFA delta), length of intensive care and hospital stay and death from any cause in the ICU or hospital.

Pharmacokinetic endpoints included the proportion of patients with detectable fludrocortisone levels and the plasma concentrations achieved at 3 hours after dosing. This interval was chosen based on prior published data in critically ill patients [9].

All outcomes were censored at 28 days or hospital discharge, whichever was earlier.

Definitions of the secondary outcomes are provided in ESM, Table S5.

### Safety outcomes

Safety outcomes included frequency of hypo- and hyperkalaemia, hypo- and hypernatremia, cumulative fluid balance and new infections.

Definitions of the safety outcomes are provided in ESM, Table S6.

### Statistical analysis

The Statistical Analysis Plan was written by study investigators prior to data analysis and database lock [12].

An independent data and safety monitoring committee (DSMC) met to review safety data after the first 100 patients had completed their primary outcome follow up. No alpha was spent to assess early efficacy; therefore, the significance threshold remained at 5% for analysis.

The primary outcome of time to resolution of shock was summarized using cumulative incidence functions treating mortality as a competing risk. Medians and quartiles of time to discharge were obtained from the cumulative incidence functions. The effect of the intervention was estimated as the hazard ratio (HR) and its 95% confidence interval (CI) obtained from a Cox model of the cause-specific hazard which estimates the chance of shock resolution in subjects who are still alive and still in shock. A pre-specified adjusted analysis of the primary outcome was performed by adding sex and the Acute Physiology and Chronic Health Evaluation (APACHE) II score as a continuous variable to the main Cox model. Other planned methods of analysing the primary outcome included examining the cumulative vasopressor dose by intervention arm and by fludrocortisone concentrations. Due to the study being stopped early, and a significant attrition in the number of patients getting samples for fludrocortisone at 3 h at the time of writing the statistical analysis plan, it was decided that a single primary endpoint was appropriate: time to shock resolution. In this report we used the vasoactive inotrope score [13], a weighted measure of pharmacological cardiovascular support) to assess vasopressor dosing and reported the comparison between groups in the secondary outcomes.

Secondary clinical outcomes of a binary nature were summarised using numbers and report proportions by treatment arm. Pairwise differences in proportions between each intervention arm and the control were calculated together with exact 95% confidence intervals using the score statistic and *p* values from Fisher exact tests. Duration outcomes (hospital stay, ICU stay and mechanical ventilation) were analysed as the number of days alive and free of outcome which was calculated between randomisation and Day 28 and summarised using means, standard-deviation, median, quartiles, minimum and maximum and compared between treatment groups using a *t* test. For continuous outcomes including change in SOFA score, maximal SOFA score, days alive and free from mechanical ventilation, days alive and free from ICU, days alive and out of hospital, mean differences between treatment groups were obtained using linear model (normal distribution and identity link). Model for maximal SOFA score included baseline measurement as one of the covariates in the model. For all hazard ratios or risk differences or mean differences, to maintain the family-wise error rate at 5% (two sided), we used a hierarchical approach by testing from the highest to the lowest dose. Only if the test for the 200 mcg arm vs control was significant at 5%, we proceeded to testing the 100mcg arm. Similarly, only if the test for the 100 mcg arm vs control was significant at 5%, did we then proceed to testing the 50 mcg arm. If testing for 200 mcg was not significant, *p* values for the other two doses were not reported, only the 95% CIs. Proportions of patients with hypokalaemia, hyperkalaemia, hyponatraemia, hypernatraemia or new onset of infection were calculated and compared across treatment arms using Fisher’s exact test.

Plasma concentrations at baseline and at three hours were described using standard summary statistics for each of the fludrocortisone dosing groups.

To account for multiple dosing at different time intervals for the three doses and, therefore, timing dependent, rather than from a single dose of each on a given day, we have plotted our data on the expected multiple dosing predicted plasma concentration–time profiles along with the known pharmacokinetics for a 50 mcg dose of fludrocortisone.

The influence of the pre-dose levels on the 3 h plasma FC concentration were adjusted for by accounting for the elimination time-course of the pre-dose using a mean terminal half-life of fludrocortisone of 2.88 h based on published data [6–8]**.** Differences between the adjusted values between the three dosing groups were analysed using a Kruskal–Wallis test.

Analysis was conducted on an intention-to-treat basis with no imputation for missing primary outcome data. All analyses were conducted primarily using SAS Enterprise Guide (version 7.1 or above).

An initial study population of 300 participants with 75 participants into each arm was planned to provide sufficient data on the fludrocortisone absorption, feasibility and to determine an efficacy signal that would inform the design of a larger trial with a mortality-based primary outcome. Assuming a shock reversal rate of 80% based on the ADRENAL [2] dataset in the hydrocortisone group, if each dose of fludrocortisone provided another 3–5% incremental shock reversal rate, the planned 300 patient study would have 70–80% power to test the trend of shock reversal across the four groups.

## Results

Due to funding constraints and slow recruitment during the coronavirus disease 2019 (COVID-19) pandemic, and after discussion with the DSMC, the Trial Steering Committee decided to stop enrolment in April 2023.

Between April 2021 and April 2023, 422 patients were screened, of whom 155 were randomised at nine sites in Australia. Of the 155 patients enrolled, consent was not obtained in two and they were excluded from the analysis. From 153 enrolled patients, 38 (24.8%) received hydrocortisone alone, 42 (27.4%) received 50 µg fludrocortisone, 36 (17.6%) received 100 µg fludrocortisone and 37 (24.1%) received 200 µg fludrocortisone (ESM, Figure S1).

Baseline characteristics of the patients are presented in Table 1. The four groups were similar at baseline with respect to primary sites of infection, illness and shock severity and distribution of severe hepatic SOFA scores although there were some differences between the groups with respect to sex distribution, the proportions of medical and surgical admission diagnoses and frequency of bacteraemia. One-hundred and one out of one-hundred and fifty-three (66%) patients had a lactate greater than 2 mmol/l prior to randomisation, thus meeting Sepsis-3 shock criteria.

**Table 1:** Baseline characteristics, collected from the 24 h prior to randomisation

Characteristics	Dose of fludrocortisone				
	0 µg (*N* = 38)	50 µg (*N* = 42)	100 µg (*N* = 36)	200 µg (*N* = 37)	Total (*N* = 153)
Age, years	64.8 ± 13.9	56 ± 14.8	60.6 ± 14.8	61 ± 14.4	60.5 ± 14.7
Female	11 (28.9%)	11 (26.2%)	12 (33.3%)	14 (37.8%)	48 (31.4%)
Weight, kg	85.5 (79; 105)	86 (75; 95)	82.5 (78.5; 100)	80 (66; 104)	85 (75; 100)
Admission source					
Emergency Department	13 (34.2%)	13 (31%)	10 (27.8%)	11 (29.7%)	47 (30.7%)
Hospital Ward	6 (15.8%)	6 (14.3%)	4 (11.1%)	5 (13.5%)	21 (13.7%)
Transfer from another ICU	6 (15.8%)	4 (9.5%)	3 (8.3%)	0 (0%)	13 (8.5%)
Transfer from another hospital	2 (5.3%)	3 (7.1%)	5 (13.9%)	1 (2.7%)	11 (7.2%)
Admitted from OR following emergent surgery	8 (21.1%)	15 (35.7%)	11 (30.6%)	15 (40.5%)	49 (32%)
Admitted from OR following elective surgery	3 (7.9%)	1 (2.4%)	3 (8.3%)	5 (13.5%)	12 (7.8%)
Primary diagnosis at ICU admission					
Medical	27 (71.1%)	26 (61.9%)	22 (61.1%)	17 (45.9%)	92 (60.1%)
Surgical	11 (28.9%)	16 (38.1%)	14 (38.9%)	20 (54.1%)	61 (39.9%)
Bacteraemia^a^	16/38 (42.1%)	14/42 (33.3%)	10/36 (27.8%)	12/37 (32.4%)	52/153 (34%)
APACHE II score	25.5 (20; 30)	23 (19; 30)	23 (19; 32.5)	25 (19; 30)	24 (19; 30)
SOFA score	11 (9; 12)	11 (9; 14)	11 (9; 13)	10 (9; 14)	11 (9; 13)
Therapies at baseline					
Invasive Ventilation	34 (89.5%)	41 (97.6%)	34 (94.4%)	36 (97.3%)	145 (94.8%)
Renal replacement therapy	5 (13.2%)	5 (11.9%)	4 (11.1%)	3 (8.1%)	17 (11.1%)
Inotropes/vasopressors					
Noradrenaline	38 (100%)	42 (100%)	36 (100%)	37 (100%)	153 (100%)
Adrenaline	9 (23.7%)	8 (19%)	8 (22.2%)	10 (27%)	35 (22.9%)
Dopamine	3 (7.9%)	3 (7.1%)	1 (2.8%)	1 (2.7%)	8 (5.2%)
Dobutamine	5 (13.2%)	0 (0%)	2 (5.6%)	5 (13.5%)	12 (7.8%)
Metaraminol	3 (7.9%)	6 (14.3%)	2 (5.6%)	3 (8.1%)	14 (9.2%)
Vasopressin	21 (55.3%)	28 (66.7%)	23 (63.9%)	22 (59.5%)	94 (61.4%)
Milrinone	0 (0%)	1 (2.4%)	0 (0%)	1 (2.7%)	2 (1.3%)
Vasoactive Inotropic Score^b^	27.3 (20; 42.3)	36.6 (24.2; 54)	29.2 (22; 45.2)	28.7 (21; 58.7)	31 (21; 50)
Time from start of inotrope/vasopressor to randomisation, hours	13.5 (7; 23)	17 (9; 35)	12.5 (7.5; 20)	16 (9; 26)	15 (8; 25)
Time from start of hydrocortisone to randomisation, hours	8 (2; 16)	8 (3; 15)	10 (3; 16)	7 (1; 15.5)	8 (3; 15)
Highest heart rate, bpm	126 ± 25	124 ± 30	121 ± 23	125 ± 27	124 ± 26
Lowest Mean arterial pressure, mmHg	58 (48; 63)	54.5 (47; 63)	58.5 (53; 62.5)	56 (51; 62)	57 (49; 62)
Lowest PaO_2_/FiO_2_ ratio, mmHg	127.5 (97; 216)	149 (101; 190)	127 (91; 183)	176 (121; 213)	146 (94; 205)
Lowest arterial PaCO_2_, mmHg	34.5 ± 7.6	33.2 ± 7.4	33.8 ± 6.8	33.4 ± 6.5	33.7 ± 7.1
Highest arterial lactate, mmol/L	3.1 (1.6; 5.6)	2.8 (1.6; 4.1)	3.2 (1.9; 6)	3.3 (1.6; 5.7)	3.1(1.8; 4.8)
Highest arterial lactate > 2 mmol/L	25 (65.8%)	26 (61.9%)	24 (66.7%)	26 (70.3%)	101 (66%)
Last serum sodium, mmol/L	138.6 ± 6.2	136 ± 4.7	137.6 ± 5.8	137.7 ± 6.5	137.5 ± 5.8
Last serum potassium, mmol/L	4.2 ± 0.6	4.2 ± 0.5	4.2 ± 0.7	4.1 ± 0.5	4.2 ± 0.6
Highest serum creatinine, umol/L	177.5 (127; 274)	197.5 (111; 275)	138 (103.5; 192.5)	169 (87; 224)	169 (108; 257)
Highest bilirubin, umol/L	20 (11; 38)	25 (13; 39)	23 (15; 42.5)	17 (10; 39)	21 (12; 39)
Lowest haemoglobin, g/L	105.2 ± 26.5	99.5 ± 21.2	99.9 ± 23.9	98.4 ± 21.3	100.7 ± 23.2
Lowest platelet count, × 10^9^/L	192 (121; 273)	131 (64; 238)	202.5 (103.5; 315)	138 (87.5; 249)	162 (89; 268)
Worst Glasgow Coma Score (non-sedated)	14 (13; 15)	14 (14; 15)	14 (14; 15)	15 (12; 15)	14 (13; 15)

*APACHE* Acute Physiology And Chronic Health Evaluation, *ICU* intensive care unit

Data expressed as *n* (%), mean ± standard deviation or median and interquartile range (Q1; Q3)

^a^Bacteraemia rate calculated from positive blood cultures related to the initial infection

^b^Vasoactive Inotropic Score calculated from the weighted sum of the maximal dose of each vasopressor/inotrope administered over the 24 h prior to randomisation

The three most common sites of infection were pulmonary, blood, and urinary with 52/153 (33.9%) of patients having positive blood cultures. The sites of infection and the pathogens are described in detail in ESM, Table S7.

### Trial and concomitant regimens

All study patients received treatment according to their allocated regimen. Sixty-nine percent of the cohort assigned to received fludrocortisone received the intervention within 1 h of randomisation. The median (interquartile range, IQR) daily doses of fludrocortisone delivered were 50 (50; 50), 100 (50; 100) and 200 (100; 200) µg in the 50 µg, 100 µg and 200 µg groups, respectively. Compliance with study drug administration was 98%, 94% and 94% in the 50 µg, 100 µg and 200 µg groups, respectively (ESM, Table S8). No patients in the hydrocortisone only group received fludrocortisone.

### Haemodynamic data

Mean arterial pressure (MAP), and heart rate between days 1 and 8 for the four groups are shown in ESM, Figure S2a and S2b. There was no between group differences in the MAPs achieved. There was statistically significantly higher heart rate over time in the 200 µg fludrocortisone group; mean difference + 8.6 bpm (95% CI 5.2–12).

### Primary outcome

**Fig. 1:**
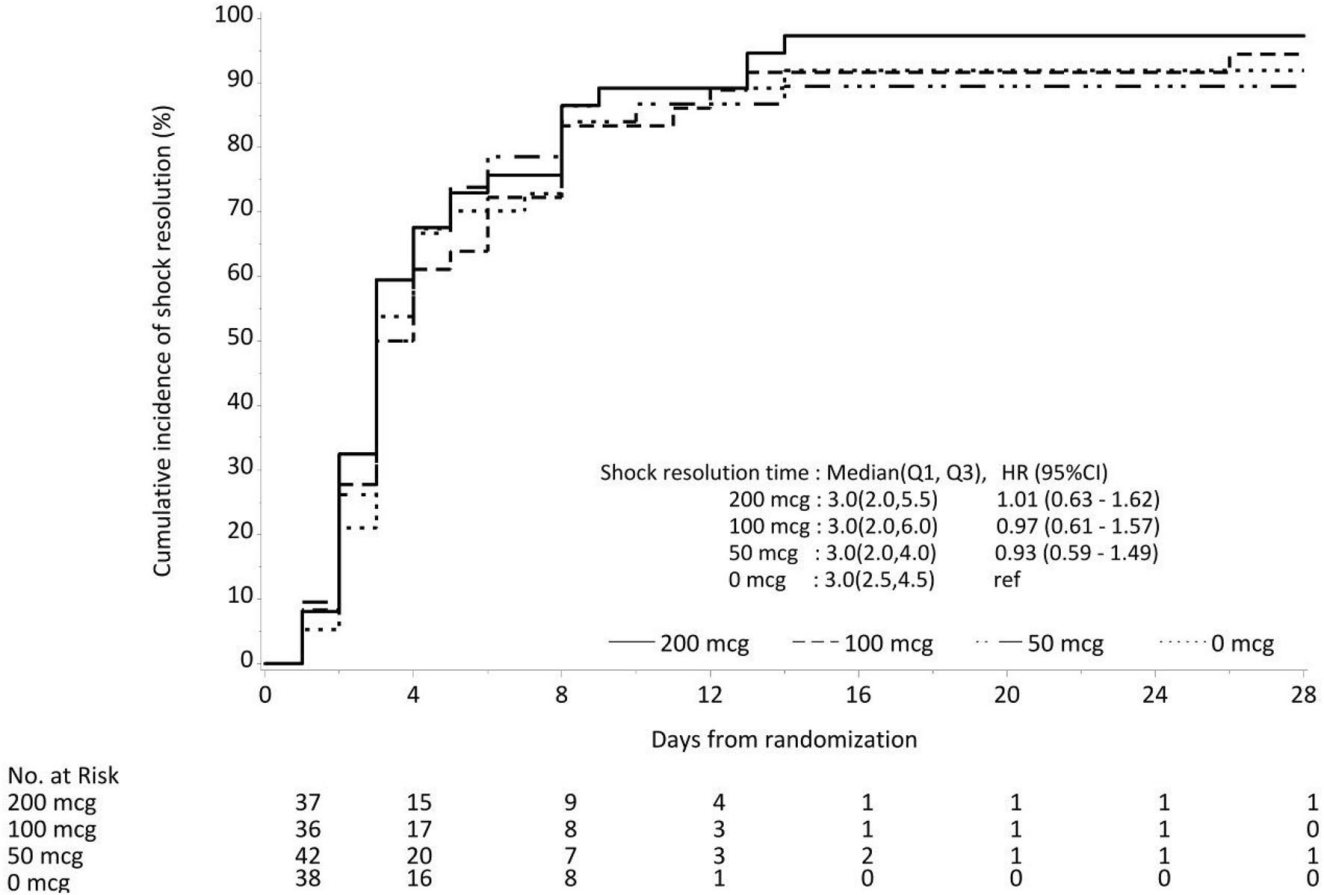
Cumulative incidence plot of shock resolution for groups according to dose of fludrocortisone received. The dotted line represents no fludrocortisone group, the dotted and interrupted lines—50 mcg group, the interrupted lines 100 mcg group and the continuous line 200 mcg group

There was no difference in the median (IQR) times to resolution of shock which were 3 (2–4.5), 3 (2–4), 3 (2–6) and 3 (2–5.5) days in the hydrocortisone alone, 50 µg 100 µg and 200 µg fludrocortisone groups, respectively, (Fig. 1 and Table 2). The hazard ratio for time to shock resolution when compared to the hydrocortisone alone group was 0.93 (95% CI 0.59–1.49), 0.97 (95% CI 0.61–1.57) and 1.01 (95% CI 0.63–1.62) in the 50 µg, 100 µg and 200 µg groups. This result did not differ when corrected for baseline sex and APACHE II scores (see Table 2).

**Table 2:** Secondary outcomes

	Fludrocortisone				Hazard ratio^A^, Absolute risk diff.^Φ^, or Mean diff. ^Γ^		
	0 µg (*N* = 38)	50 µg (*N* = 42)	100 µg (*N* = 36)	200 µg (*N* = 37)	50 µg	100 µg	200 µg
Median time to resolution of shock (IQR), days ^A^	3 (2.5; 4.5)	3 (2; 4)	3 (2; 6)	3 (2; 5.5)	0.93 (0.59, 1.49)	0.97 (0.61, 1.57)	1.01 (0.63, 1.62) p = 0.96
Time to resolution of shock-adjusted model ^A^ ^a^					0.93 (0.58, 1.49)	0.93 (0.58, 1.51)	1.07 (0.66, 1.71) p = 0.79
28 day resolution of shock—no./total no. (%) ^ɸ^	28/38 (73.7)	33/42 (78.6)	32/36 (88.9)	32/37 (86.5)	– 0.02 (– 0.14, 0.11)	0.05 (– 0.05, 0.15)	0.05 (– 0.05, 0.15) p = 0.61
Recurrence of shock—no./total no. (%) ^ɸ^	9/38 (23.7)	11/42 (26.2)	6/36 (16.7)	10/37 (27)	0.03 (– 0.16, 0.21)	– 0.07 (– 0.25, 0.11)	0.03 (– 0.16, 0.23) p = 0.80
28 day mortality—no./total no. (%) ^ɸ^	9/38 (23.7)	7/42 (16.7)	4/36 (11.1)	4/37 (10.8)	– 0.07 (– 0.25,0.11)	– 0.13 (– 0.3,0.04)	– 0.13 (– 0.30, 0.04) p = 0.22
Median days to mortality (IQR)—days ^A^	11 (8; 12)	9 (7; 18)	7.5 (4.5; 13.5)	14 (10.5; 17)	0.7 (0.26, 1.88)	0.48 (0.15, 1.57)	0.43 (0.13, 1.4) p = 0.16
New infection requiring antibiotics—no./total no. (%) ^ɸ^	7/38 (18.4)	6/42 (14.3)	7/36 (19.4)	6/37 (16.2)	– 0.04 (– 0.2, 0.12)	0.01 (– 0.17, 0.19)	– 0.02 (– 0.19, 0.15) p > 0.99
Days alive and free from mechanical ventilation ^Γ^	20.5 ± 5.39	20.3 ± 6.75	20.5 ± 6.9	19.4 ± 7.94	– 0.26 (– 3.27, 2.74)	– 0.06 (– 3.22, 3.11)	– 1.11 (– 4.23, 2.01) p = 0.48
Delta SOFA ^Γ^	− 0.6 ± 2.73	− 0 ± 2.69	-0.6 ± 2.39	0.4 ± 2.73	0.58 (– 0.58, 1.75)	0.02 (– 1.19, 1.23)	1.01 (– 0.2, 2.22) p = 0.1
Maximum SOFA ^Γ^	10.7 ± 3.34	11.3 ± 4.15	10.3 ± 3.03	11.1 ± 3.13	0.58 (– 0.55, 1.72)	– 0.09 (– 1.27, 1.09)	0.87 (– 0.31, 2.04) p = 0.14
Median time to first discharge from the ICU (IQR)—days ^A^	8 (5; 12)	7 (6; 13)	7.5 (4; 14)	9 (4; 15)	0.83 (0.5, 1.38)	0.96 (0.58, 1.59)	0.82 (0.49, 1.36) p = 0.44
No. of days alive and out of the ICU ^Γ^	18.6 ± 5.75	17.3 ± 7.87	17.3 ± 8.46	16.9 ± 7.96	– 1.24 (– 4.59, 2.11)	– 1.25 (– 4.73, 2.23)	– 1.69 (– 5.14, 1.77) p = 0.34
Median time to discharge from the hospital (IQR)—days ^A^	23 (12; 28)	26 (14; 28)	28 (10.5; 28)	21 (15; 28)	0.88 (0.46,1.69)	0.88 (0.45, 1.72)	1.29 (0.69, 2.41) p = 0.42
No. of days alive and out of the hospital ^Γ^	3.6 ± 6.2	4.7 ± 7.6	5.3 ± 8.4	5.9 ± 6.8	1.06 (– 2.17, 4.28)	1.7 (– 1.65, 5.05)	2.29 (– 1.04, 5.61) p = 0.18

*ICU* intensive care unit, *IQR* interquartile range, *SOFA* sequential organ failure assessment

^A^ Hazard ratios (95% CI). Hazard ratio and its 95% confidence interval obtained from a Cox model of the cause-specific hazard which estimates the risk of event of interest in subjects who are still alive and have not yet experienced the event

^ɸ^Risk difference (95% CI). Pairwise differences in proportions between each intervention arm and the control were calculated with exact 95% confidence intervals using the score statistic and *p* values from Fisher’s exact test

^Γ^Mean difference (95% CI). For continuous outcomes including: change in SOFA score, maximal SOFA score, Days alive and free from mechanical ventilation, no. of days alive and free from ICU, no. of days alive and out of Hospital, mean differences between treatment groups were obtained using linear model (normal distribution and identity link). Model for maximal SOFA score included baseline measurement as one of the covariates in the model

^a^Hazard ratio and 95% confidence interval obtained from Cox model. Adjusted model adjusted for sex and baseline APACHE score

*P* values are shown at the bottom of each cell under the Hazard Ratio or Risk difference or Mean difference. To maintain the family-wise error rate at 5% (two sided), we used a hierarchical approach by testing from the highest to the lowest dose. Only if the test for the 200 mcg arm vs control is significant at 5%, we proceeded to testing the 100 mcg arm, otherwise we will stop testing. Likewise, only if the test for the 100 mcg arm vs control was significant at 5%, will we then proceed to testing the 50 mcg arm. If testing for 200 mcg was not significant, we have not reported *P* values for the other two doses, only the 95% CIs

Two post hoc sensitivity analyses were conducted with respect to the primary outcome—in the subgroup of patients meeting Sepsis-3 criteria in the various fludrocortisone groups and combining all fludrocortisone groups and comparing to hydrocortisone alone. There was no evidence of a differential treatment effect in the subgroup of patients meeting Sepsis-3 criteria in the various fludrocortisone groups—50 mcg [HR 0.90 (95% CI: 0.50–1.63)], 100 mcg [HR 1.05 (95% CI: 0.59–1.91)] and 200 mcg [HR 0.97 (95% CI: 0.55–1.73].

Combining all fludrocortisone groups and comparing to hydrocortisone alone did not demonstrate evidence of a treatment effect with respect to the primary outcome [HR 0.97 (95% CI 0.66–1.43) *P* = 0.89].

### Secondary outcomes

The secondary outcomes are reported in Table 2.

The proportions of patients alive and experiencing resolution of shock at 28 days were 28/38 (73.7%), 33/42 (78.6%), 32/36 (88.9%) and 32/37 (86.5%) in the hydrocortisone alone, 50 µg 100 µg and 200 µg fludrocortisone groups, respectively. Shock recurrence occurred in 9/38 (23.7%), 11/42 (26.2%), 6/36 (16.7%) and 10/37 (27%) of patients in the hydrocortisone alone, fludrocortisone 50 µg, 100 µg and 200 µg groups, respectively. The vasoactive-inotropic score (VIS) was statistically significantly higher in the 50 µg group as compared to control with a mean difference 12.96 (95% CI 0.38–25.55); however, this effect was not apparent in the 100 and 200 µg groups (ESM, Figure S2c).

The cumulative incidence functions of time to cessation of mechanical ventilation, ICU discharge and hospital discharge did not differ between the groups (ESM, Figs S3a-S3c).

The SOFAmax and SOFA delta within days 1–8 did not differ between groups.

The 28-day mortality rate was 23.7% (9/38) in the hydrocortisone alone group compared to 16.7% (7/42), 11.1% (4/36) and 10.8% (4/37) in the 50 µg, 100 µg and 200 µg groups, respectively.

### Safety outcomes

The four groups did not differ in the frequency of secondary safety outcomes which included rates of abnormalities of sodium and potassium, assessment of fluid balance and incidence of new infections (Table 3). Furthermore, groups did not differ in daily laboratory data and measures of organ dysfunction (see ESM, Table S10).

**Table 3:** Safety outcomes

	Fludrocortisone				*P* value
	0 µg	50 µg	100 µg	200 µg	
Hypernatraemia (> 150 mmol/l) (%)	29	19	22	30	0.64
Hyponatraemia (< 135 mmol/l) (%)	44	59	47	50	0.63
Hyperkalaemia (> 5.0 mmol/l) (%)	18	31	25	30	0.58
Hypokalaemia (< 3.5 mmol/l) (%)	56	66	57	58	0.81
Daily fluid balance Median (IQR) mls	53 (− 1104; 1094)	7 (− 1108; 1007)	368 (− 736; 1279)	102 (− 989; 1040)	0.05
New infection (%)	18	14	19	16	0.94

*IQR* interquartile range

### Plasma fludrocortisone concentrations

**Fig. 2:**
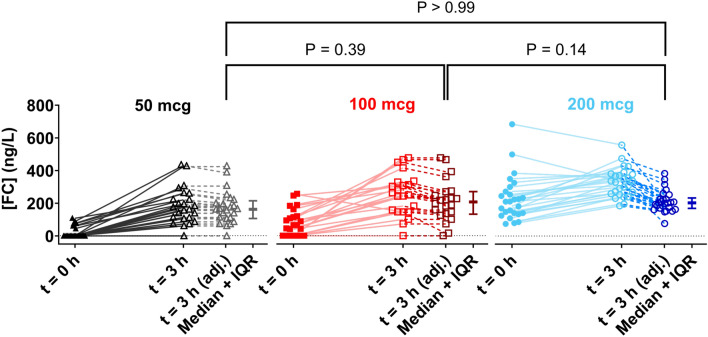
Visual depiction of FC plasma levels at pre-dosing (0 h) (closed symbol) and 3 h (open symbol) after FC dosing for the FC dosing regimens populations for 50 (), 100 () and 200 () mcg. Also shown are the 3 h values for each dosing regimen after adjusting for elimination of their pre-dose over the 3 h dosing interval (), along with the median ± IQR for each group. No significant difference between the adjusted values was found with a Kruskal–Wallis test (*p* > 0.05)

Of the 115 patients receiving fludrocortisone, 74 had fludrocortisone concentration measured post dose. 97% (72/74) patients had detectable plasma fludrocortisone concentrations at 3 h post dose. The median [IQR] plasma concentrations were 261 ng/L [156–334], with a wide range 0–677 ng/L. Figure 2 illustrates the adjusted values for plasma fludrocortisone concentrations after accounting for the pre-dose levels and drug elimination. No significant difference in FC plasma levels at 3 h post-study dose is observed between dosing groups following the correction for pre-dose levels (*P* > 0.05). A plot of our data on the expected multiple dosing predicted plasma concentration–time profiles for 50mcg multiple dosing, equivalent to daily doses of 50, 100 and a 200 mcg dose of fludrocortisone and previously published data on pharmacokinetics of fludrocortisone are shown in ESM, Figure S4.

There was no significant correlation between plasma fludrocortisone concentrations at time 0 or 3 h and the VIS scores (ESM, Figure S5).

### Adverse events and protocol deviations

There were no serious adverse events. Two adverse events occurred, details of which are provided in ESM, Table S9. Seven ineligible patients were randomised. One was receiving a daily total dose of 300 mg rather than 200 mg hydrocortisone and the remaining 6 patients all received hydrocortisone after, rather than prior to study inclusion.

## Discussion

In this phase II study, we found that plasma fludrocortisone was detected in the majority of critically ill patients with septic shock following enteral administration. The concomitant administration of fludrocortisone at different doses with hydrocortisone did not result in faster shock reversal than hydrocortisone alone. There were no differences between the groups with respect to recurrence of shock, organ failure scores, duration of mechanical ventilation, mortality and length of ICU and hospital stay. The higher vasopressor requirement in the 50 µg group compared to the 0 µg group contrasts with non-significantly different vasopressor requirements in the 100 µg and 200 µg groups and, given multiple comparisons, this result should be considered exploratory.

This study provides new evidence about the absorption of enterally administered fludrocortisone in critically ill patients with 97% of the study cohort demonstrating absorption of enterally administered fludrocortisone. In contrast to previous, smaller studies, the assay used in this study had increased sensitivity with a lower level of detection of 40 ng/L compared to 100 ng/L [9]. The wide variability in plasma fludrocortisone concentrations observed in our study, however, suggests differing absorption and bioavailability.

The differences in pre-dose concentrations are probably a function of the time interval between the doses. The predicted pharmacokinetics of fludrocortisone as determined by the dosing regime, associated with the participant randomisation into the study (ESM, Figure S4), demonstrates that the observed plasma concentration of fludrocortisone in patients, as viewed as a population, had no systematic bias either above or below the predicted fludrocortisone plasma concentration once the dosing regime and sampling time are considered. Fludrocortisone is predominantly metabolised in the liver. However, altered liver metabolism is unlikely to have contributed to the profile of serum fludrocortisone concentrations in the 3 groups as the proportion of patients with severe hepatic scores at baselines and changes in the delta SOFA and maximum SOFA were similar across the three fludrocortisone groups.

Prior studies of fludrocortisone in septic shock patients have used once daily dosing [3, 9, 14]. Pharmacokinetic and pharmacodynamic studies in healthy humans have suggested more frequent dosing should be considered [15]. In a trial including 12 participants, the plasma fludrocortisone level that achieved 50% of the pressor response was modelled to be 268 ng/l [16].

We did not observe any serious adverse effects attributable to fludrocortisone use. An increased rate of superinfection was reported in the Corticosteroid and Intensive Insulin Therapy for Septic Shock (COIITTS) study in patients assigned to the fludrocortisone arm [14] which we did not replicate.

Fludrocortisone is a potent mineralocorticoid and exerts it effects through the mineralocorticoid receptor. Whether alterations in receptor gene expression influence response to fludrocortisone is unclear. Whilst data from murine septic shock models and in vitro data using human endothelial and smooth muscle cells suggest blunted mineralocorticoid receptor expression as a potential cause of hemodynamic failure [17], data from a large nested cohort study of septic shock patients suggest no relationship between either plasma aldosterone [18] or mineralocorticoid receptor gene expression [19] and mortality or reversal of shock.

Our trial was designed using the modified ADRENAL [2] inclusion criteria with statistical power to detect a clinically plausible effect of fludrocortisone on reversal of shock. To reduce bias, we used a central randomisation process and ensured the concealment of trial-group assignments. We published our statistical analysis plan before database lock. As this was a dose finding study, we chose shock resolution as the primary outcome and specifically targeted a population of patients who had high requirements for vital organ support (use of mechanical ventilation, vasopressor therapy and need for hydrocortisone). The baseline illness severity and organ failure scores, and the high need for organ support, suggest we enrolled the appropriate target population. A high proportion of eligible patients received the trial intervention as planned, and none were lost to follow-up.

Our trial had limitations. Our study was underpowered to detect differences in shock reversal owing to premature termination of the trial for logistic reasons. As this was a pragmatic trial, we did not protocolise haemodynamic targets or vasopressor wean. We used Sepsis-2 [20] definitions in accordance with the ADRENAL trial, but based upon baseline lactate levels, nearly 66% of patients met Sepsis-3 criteria [21]. In this phase II study, we initially planned co-primary outcomes but later refined this to a single primary outcome as per our statistical analysis plan. The primary outcome was not patient centred. The administration of interventions was unblinded and may have influenced the assessment of the primary outcome. However, our results are in accord with a previous larger trial reporting similar vasopressor free days in patients assigned to hydrocortisone vs hydrocortisone plus fludrocortisone [14]. We only reported the relationship between fludrocortisone concentrations and VIS scores, a detailed pharmacokinetic–pharmacodynamic analysis would have been more informative. Within the context of an open label trial, we collected data on only adverse events that had been judged by the treating clinicians to be related to the trial regimen, and we did not adjudicate this assessment.

Our data inform clinicians regarding the absorption, and safety of different doses of fludrocortisone when planning a phase III trial of fludrocortisone in septic shock.

## Conclusion

In conclusion, in critically ill mechanically ventilated patients with septic shock, following enteral administration of fludrocortisone, we found detectable plasma concentrations of fludrocortisone in the majority of patients, although plasma concentrations varied widely indicating differing absorption and bioavailability. The addition of fludrocortisone to hydrocortisone was not associated with faster shock resolution.

## Supplementary Information

Below is the link to the electronic supplementary material.

Supplementary file1 (DOCX 2567 KB)
