# Supplementary material for: Fludrocortisone dose–response relationship in septic shock: a randomised phase II trial
Source: Intensive Care Med. 2024 Sep 5;50(12):2050–60. doi: 10.1007/s00134-024-07616-z (PMC11588801; doi:10.1007/s00134-024-07616-z)
Supplement: Supplementary file 1 — Supplementary file1 (DOCX 2567 KB) [file 134_2024_7616_MOESM1_ESM.docx]

**Supplementary Appendix for the manuscript “Fludrocortisone Dose Response Relationship in Septic Shock – A Randomised Phase II Trial”**

**Table of contents Page**

**Study management committee 2**

**Study participating sites 2**

**Data safety monitoring committee 2**

**Table 1 - Source data verification and monitoring 3**

**Table 2 - Inclusion and exclusion criteria 4**

**Table 3 - Study drug and blood testing regimen 5**

**Table 4 - Fludrocortisone assay methodology 6**

**Table 5 - Secondary outcome definitions 9**

**Table 6 - Safety outcomes 10**

**Table 7 - Sites of infection and infecting pathogens 11**

**Table 8 – Compliance to study treatment 13**

**Table 9 - Adverse events 14**

**Table 10 – Daily laboratory and organ failure data 15**

Figure 1 - Study flowchart 16

**Figure 2 – Mean plots of the lowest MAP, heart rate and 17**

**Vasoactive Inotropic Score over time**

**Figure 3 – Cumulative incidence function of time to cessation 19**

**of mechanical ventilation, ICU discharge and hospital discharge**

**Figure 4 – Plasma fludrocortisone levels at time 0 and 3 hours in 21**

**each of the 3 dosing groups.**

**Figure 5 - A plot of VIS vs plasma fludrocortisone concentrations 22**

**at time 0 and 3 hrs.**

**Study management committee**

James Walsham - Princess Alexandra Hospital, Brisbane

Bala Venkatesh - The George Institute for Global Health, Sydney; The Wesley Hospital, Brisbane

John Myburgh - The George Institute for Global Health, Sydney

Simon Finfer - The George Institute for Global Health, Sydney; Royal North Shore Hospital

Jeremy Cohen - The Royal Brisbane and Women’s Hospital; The Wesley Hospital, Brisbane

Naomi Hammond - The George Institute for Global Health, Sydney

Peter Kruger - Princess Alexandra Hospital, Brisbane

Dorrilyn Rajbhandari - The George Institute for Global Health, Sydney

Jason Meyer - Princess Alexandra Hospital, Brisbane

**Study participating sites**

1. The Princess Alexandra Hospital, Brisbane
2. The Wesley Hospital, Brisbane
3. Gold Coast University Hospital, Gold Coast
4. The Royal Brisbane and Womens Hospital, Brisbane
5. The Mater Hospital, Brisbane
6. The Royal North Shore Hospital, Sydney
7. The Austin Hospital, Melbourne
8. Blacktown Hospital, Sydney
9. The Queen Elizabeth Hospital, Adelaide

**Data safety monitoring committee**

Prof Andrew Udy - Alfred Hospital; ANZIC Research Centre, Melbourne

Prof Michael Stowasser – University of Queensland Southside clinical school, Brisbane

**Table S1 - Source data verification and monitoring**

Source data verification (SDV) and monitoring activities were performed at each site and remotely by a study monitor or the study Project Manager.

The monitor ensured that the study was being conducted according to the protocol, good clinical practice guidelines and relevant regional regulatory requirements.

All written informed consent forms were 100% source data verified. During the Covid-19 pandemic, the ethics committee gave approval for verbal consent, where written consent was not possible, and the documentation of this process was source data verified

Changes to Source Data Verification and Monitoring due to the COVID-19 Pandemic

From February 2021 until June 2021, monitoring activities were significantly affected by the COVID-19 pandemic. This was due to pandemic planning in participating Intensive Care Units, including redeployment of research staff to clinical roles, travel restrictions, infection control measures, and visitor restrictions.

Due to limitations imposed by the COVID-19 Pandemic and budgetary restraints, no other source data verification was performed at sites during the study. 100% participant data was reviewed remotely on the secure web-based study database, applying validation and consistency rules. Query resolution and regular data cleaning were undertaken to confirm data consistency and integrity.

**Table S2 - Inclusion and exclusion criteria**

| **Inclusion Criteria**   1. Aged 18 years or older 2. Documented site, or strong suspicion of infection with 3. 2 of the 4 clinical signs of inflammation:    1. Core temperature > 38^o^C or < 35^o^C    2. Heart rate > 90bpm    3. Respiratory rate > 20bpm, or PaCO2 < 32mmHg, or mechanical ventilation    4. White cell count > 12 x 10^9^/L or < 4 x 10^9^/L or > 10% immature neutrophils 4. Being treated with continuous vasopressors or inotropes to maintain a systolic blood pressure > 90mmHg, or mean arterial pressure > 60mmHg or a MAP target set by the treating clinician for maintaining perfusion* 5. Being treated with Hydrocortisone at a daily dose of 200mg / day as adjunctive treatment for septic shock 6. Being treated with mechanical ventilation (either invasive or non-invasive) at the time of randomisation (Non-invasive includes mask BiPAP/CPAP)   *A minimum duration of vasopressors was not mandated as all patients would need to be on hydrocortisone for septic shock prior to enrolment and as this was a pragmatic Phase-II trial it was anticipated that clinicians would only commence hydrocortisone if there was a persistent vasopressor requirement.  **Exclusion Criteria**   1. Met all inclusion criteria more than 24 hours ago 2. Patients taking long term corticosteroids or fludrocortisone 3. Patients with systemic fungal infection 4. Death is deemed inevitable or imminent during this admission and either the attending physician, patient or surrogate legal decision maker is not committed to active treatment 5. Patient unable to receive enteral medication 6. Death from underlying disease likely within 90 days 7. Patient has been previously enrolled in the study |
| --- |

**Table S3 - Study drug and blood testing regimen**

Study drug was commercially available fludrocortisone acetate. Fludrocortisone was administered enterally. Where this was via a gastric feeding tube, the drug was crushed, dissolved in water and administered via the tube with a further flush of water, according to study site local practice.

Patients randomised to receive fludrocortisone were administered their first ‘stat’ dose as soon as possible following randomisation. Subsequent dosing occurred at the following scheduled times, as per the below table. If the first schedule dose was within 3 hours of the ‘stat’ dose, then that scheduled dose was deferred until the next time due.

| Patients randomised to 50 mcg once daily fludrocortisone (total of 8 doses, including the ‘stat’ dose) | Dose at 08:00 hours |
| --- | --- |
| Patients randomised to 50 mcg twice daily fludrocortisone (total of 15 doses, including the ‘stat’ dose) | Dose at 08:00 and 20:00hours |
| Patients randomised to 50 mcg four times daily fludrocortisone (total of 29 doses, including the ‘stat’ dose) | Dose at 02:00, 08:00, 14:00 and 20:00 hours |

Patients received fludrocortisone for 7 days (maximum number of doses as per the above table) or until hydrocortisone was ceased or until discharge from ICU whichever was earlier.

To assess the plasma levels of fludrocortisone in patients receiving fludrocortisone blood samples were taken prior to, and 3 hours after drug administration. Samples were collected at a time of research staff convenience between the first scheduled drug dose and day 5.

In a cohort of 30 patients assigned to fludrocortisone (10 in each group), additional blood samples for detailed pharmacokinetic analyses were undertaken.

**Table S4 - Fludrocortisone assay methodology**

Two LCMS instruments, an earlier study^^[[1]](#footnote-1)^^ and an internal standard, fludrocortisone-d6 (FC-d6, Expert Synthesis Solutions, Fig. S4.1) were used in developing and validating an analytical method for fludrocortisone (FC, Toronto Research Chemicals, Fig. S4.1) plasma concentrations.

| 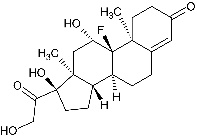 |  | 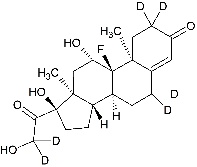 |
| --- | --- | --- |

**Fig. S4.1** Fludrocortisone (on left, FC) and fludrocortisone-d6 (on right, FC-d6) internal standard.

The two LC-MS/MS systems used to analyse the samples were of an AB Sciex API6500+® and Shimadzu 8060®a triple-quadrupole mass-spectrometer, both attached to a Shimadzu SIL-30AC-HT® dual-pump UPLC systems. Their conditions and instrumental parameters for the AB Sciex API6500+ instrument is shown in Table S4.1.

**Table S4.1a** Instrumental parameters for the AB Sciex API6500+

| **Chromatographic Conditions** | | | | | | | |
| --- | --- | --- | --- | --- | --- | --- | --- |
| Injection Volume | | 50 µL | | | Gradient Timepoints | | |
| Flow Rate: | | 0.3 mL/min | | | Time (min) | | %B |
| LC Run Time: | | 8.0 min | | |  |  |  |
| Mobile Phase A: | | 0.2 mM ammonium fluoride solution | | | 0.00 | | 40 |
| Mobile Phase B: | | 0.2 mM ammonium fluoride in 5:95 water:methanol | | | 3.50 | | 68 |
| Column: | | Kinetex® 3.0 µm C18 100 Å, LC Column 100 mm x 3 mm | | | 6.00 | | 86 |
| Guard: | | 4 mm x 3 mm C18 | | | 6.50 | | 100 |
| Column Oven: | | 40° C | | | 6.60 | | 100 |
|  | |  | | | 6.80 | | 40 |
|  | |  | | | 8.00 | | 40 |
| **Mass Spectrometer Conditions** | | | | | | | |
| Mode: | ESI | |  | Spray Voltage | | 4500 V | |
| Source Gas 1 | 60 psi | |  | Source Temperature | | 700 °C | |
| Source Gas 2 | 50 psi | |  | Mode | | Scheduled MRM | |
| Curtain Gas | 20 psi | |  | Retention Time Tolerance | | 30 sec | |
| CAD Gas | 9 psi | |  |  | |  | |

**Table S4.1b** Ion-specific parameters for the AB Sciex API6500+

| **Target** | **Mode** | **Use** | **Precursor ion (Q1)** | **Product ion (Q3)** | **MRM window (min)** | **DP (V)** | **EP (V)** | **CE (V)** | **CXP (V)** | **Dwell Time (ms)** |
| --- | --- | --- | --- | --- | --- | --- | --- | --- | --- | --- |
| FC | Positive | quantitation | 381.0 | 239.0 | 3.83 – 4.83 | 100 | 10 | 30 | 15 | 250 |
|  |  | confirmation | 381.0 | 181.0 |  | 80 | 10 | 30 | 10 | 50 |
| FC-d6 |  | quantitation | 387.0 | 243.0 | 3.83 – 4.83 | 100 | 10 | 30 | 15 | 250 |
|  |  | confirmation | 387.0 | 185.0 |  | 80 | 10 | 30 | 10 | 50 |

The overall method involved thawing 300 µL of plasma (collected in EDTA, stored in polypropylene tubes at -70°C) to room temperature, transfer to a borosilicate glass test tube and adding methanol (16.7 µL) and IS solution (15 µL), vortexing, then treating with NH_4_OH solution (0.25%, 7.5 µL) with mixing for 5 seconds. FC and FC-d6 were then extracted into methyl tert-butyl ether (MTBE, 2 mL) by an initial mixing for 5 s and shaking on a platform rocker for 10 min. The organic layer (1.4 mL) was transferred to a borosilicate glass tube, where it was evaporated at room temperature. The dry residue was redissolved in 10 mM ammonium formate with 0.1% formic acid in 50:50 water: methanol (75 µL) and centrifuged at 12,000 rpm for 10 minutes at 4°C. The supernatant was transferred to a borosilicate glass inserts in amber glass autosampler vials with PTFE/ Si septa prior to injection.

Stock solutions and quality control (QC) samples of FC were prepared in methanol and diluted and spiked into drug-free human plasma to make calibration standards with FC concentrations ranging from 25 to 1000 pg/mL. The FC-d6 internal standard was prepared as a 5 ng/mL solution in methanol. Typical chromatograms are shown in Fig. S4.2.

| 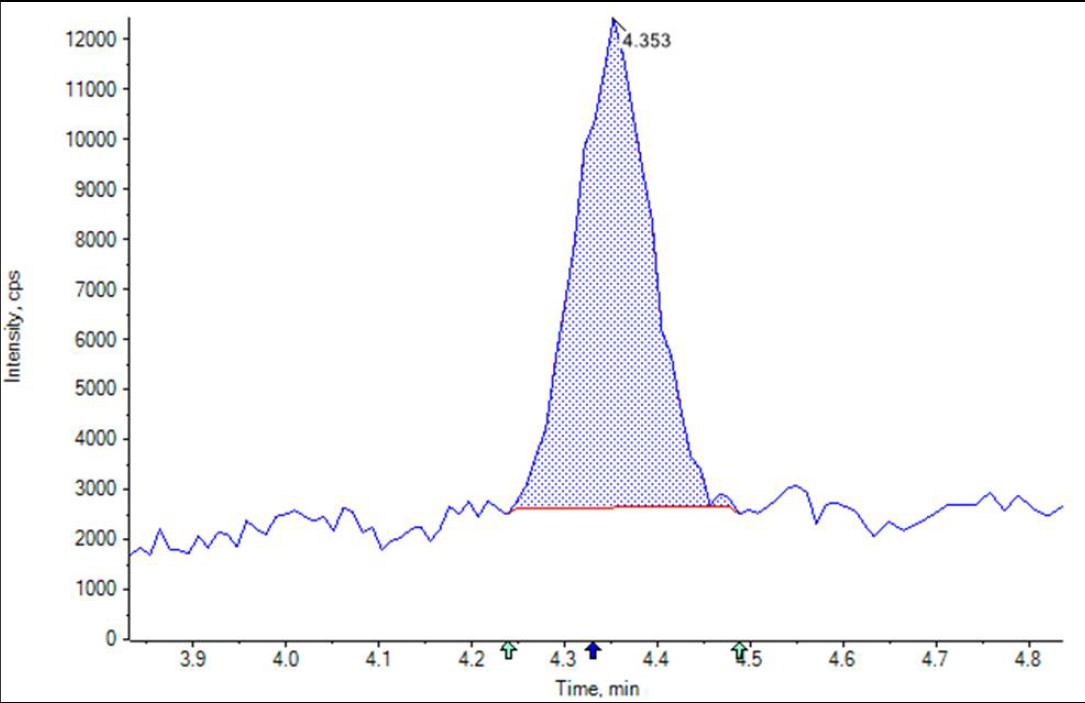  **a.** LLOQ [FC]=25ng/L | 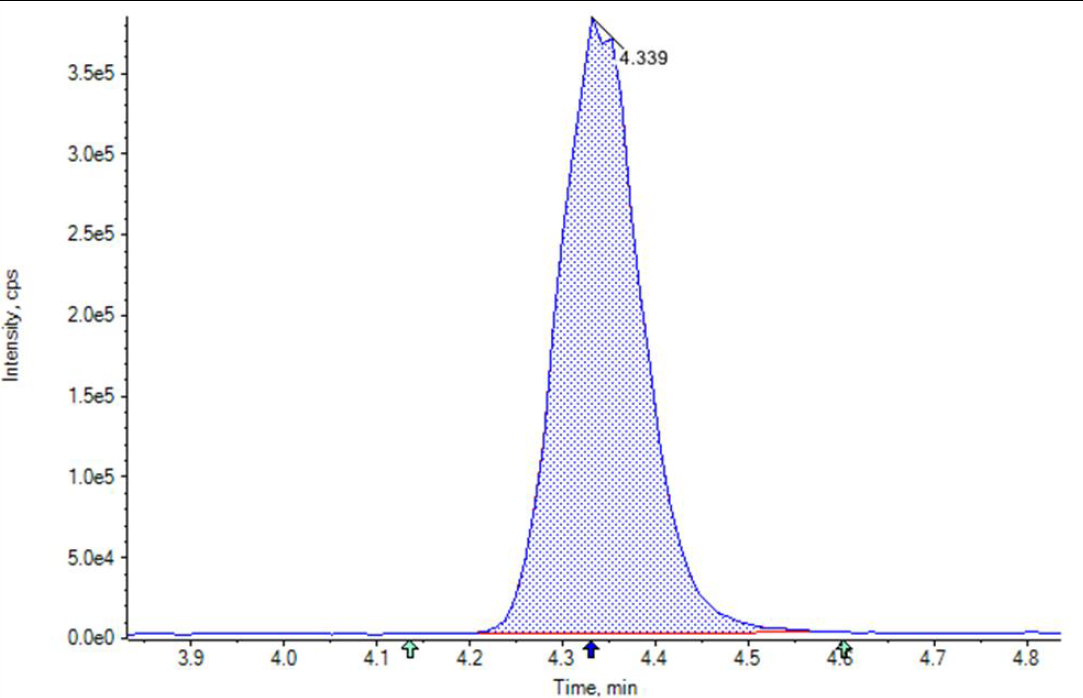  **b.** ULOQ [FC]=1000ng/L |
| --- | --- |
| 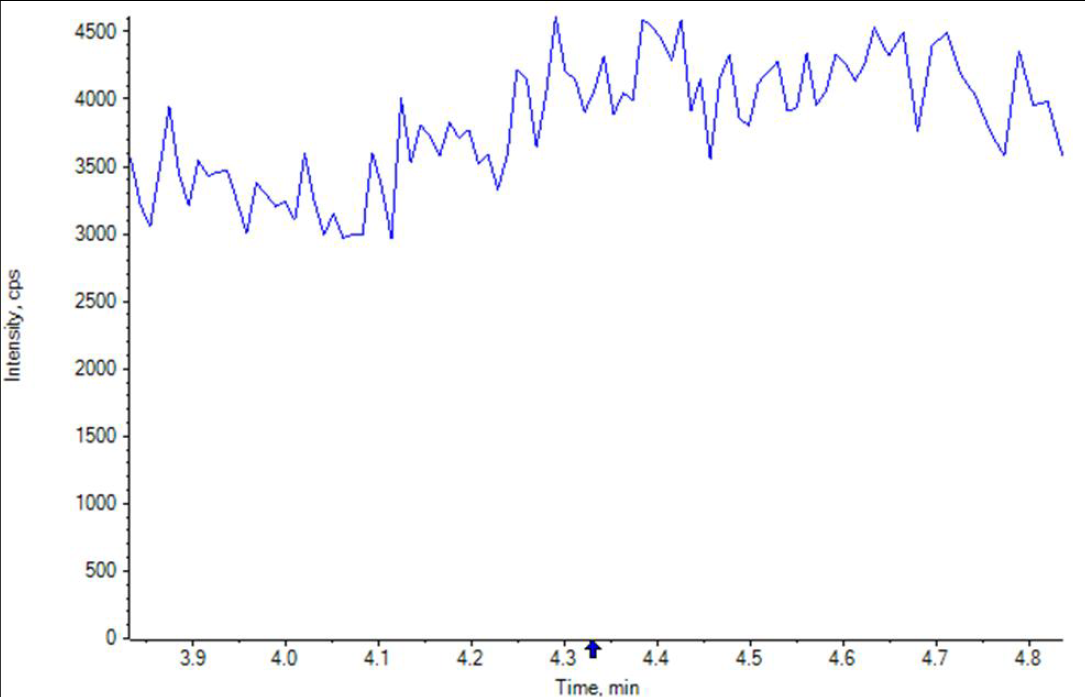  **c.** blank | 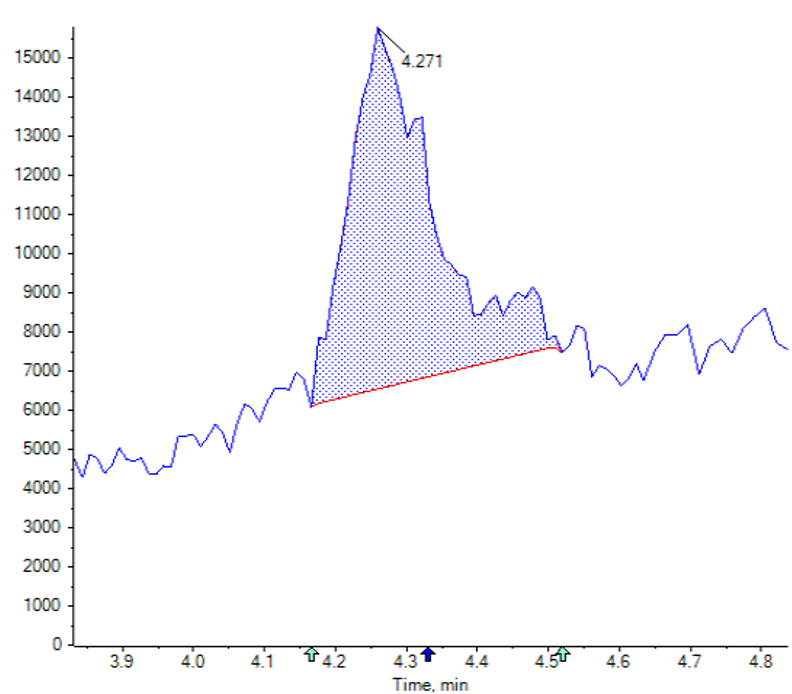  **d.** internal standard |

**Figure S4.2** Representative chromatograms of a: the lowest FC standard, b: the highest FC standard, c: blank plasma & d: the FC-d6 internal standard.

**Figure S4.3** A representative calibration curve of PAR versus FC plasma concentrations.

The fludrocortisone plasma concentrations were deduced from a quadratic regression calibration curve (with a 1/[FC]^2^ weighting) of the instrument response peak area ratio (PAR) for the chromatographic peaks of FC and FC-d_6_ (IS) using the calibration curve shown in Fig. S4.3.

The validation QC results for the AB Sciex API6500+®instrument are shown in Table S4.2. The stability QC results, undertaken, for both low and high FC concentrations. are shown in Table S4.3.

**Table S4.2** Summary of Method 1 FC QC validation accuracy and precision

| **Label** | **QC 1** | **QC 2** | **QC 3** | **QC 4** |
| --- | --- | --- | --- | --- |
| **Nominal conc. (pg/mL)** | **25** | **50** | **200** | **500** |
| **Intra-assay Accuracy** | 5.6% | -2.0% | 6.4% | 3.6% |
| **Intra-assay Precision** | 6.9% | 8.3% | 2.8% | 6.7% |
| **Inter-assay Accuracy** | 5.6% | 0.4% | 7.9% | 6.2% |
| **Inter-assay Precision** | 6.9% | 8.7% | 6.5% | 9.9% |

**Table S4.3** Summary of stability tests

|  | **QC concentration % difference** | |
| --- | --- | --- |
| **Exposure Condition** | **L** | **H** |
| **Storage of plasma in vials at ambient conditions for >18h overnight** | 34.7 | 59.7 |
| **Storage of extracts on the autosampler for >18h overnight** | 3.5 | -1.2 |
| **Three freeze/thaw cycles of plasma between -20°C and room temperature** | 34.1 | 64.2 |

**Table S5 – Secondary outcome definitions**

**Recurrence of shock** – defined as a new episode of hemodynamic instability requiring treatment with vasopressors or inotropes after resolution of the initial episode**.**

**Ventilation free days** – defined as number of days alive and free of positive pressure ventilation (invasive and non-invasive) from randomization to 28 days.

**ICU and hospital length of stay** – defined as the total duration of stay for the first 28 days after randomisation.

**ICU and 28 day mortality** – defined as death in ICU or hospital before day 28

**Delta SOFA and Maximal SOFA score** – SOFA scores were collected daily from baseline to day 8. Delta SOFA is the difference between the baseline SOFA score and the maximal SOFA score, where the maximal SOFA score is the highest SOFA score collected after randomisation.

**Pharmacokinetic outcomes**

-**Plasma levels** defined as the plasma concentration achieved at 3 hours post fludrocortisone dosing.

-**Absorption** defined as the proportion of patients with detectable fludrocortisone levels

**Table S6 – Safety outcomes**

Daily highest and lowest Sodium and Potassium and daily fluid balance were collected through to day 8.

- 1. Hyper- and Hyponatraemia was defined as sodium > 150 and sodium <135mmol/L.
  2. Hyper- and Hypokalaemia was defined as Potassium >5.0 and Potassium < 3.5mmol/l.
  3. New infection was defined by the prescription of a new antibiotic course occurring over 48hrs following commencement of study drug.

**Table S7 - Sites of infection and infecting pathogens**

|  | **Fludrocortisone ________________________________________________________________** | | | |  |
| --- | --- | --- | --- | --- | --- |
| **Documented site or strong suspicion of infection** | **0 mcg (N=38)** | **50 mcg (N=42)** | **100 mcg (N=36)** | **200 mcg (N=37)** | **Total (N=153)** |
|  | | | | | |
| **Patients with at least one confirmed primary site of infection** | 24/38 (63.2%) | 27/42 (64.3%) | 22/36 (61.1%) | 24/37 (64.9%) | 97/153 (63.4%) |
|  | | | | | |
|  | | | | | |
| **Site - Pulmonary** |  |  |  |  |  |
| Streptococcus pneumoniae (or Pneumococcus) | 0/5 (0.0%) | 2/7 (28.6%) | 1/6 (16.7%) | 1/7 (14.3%) | 4/25 (16.0%) |
| Klebsiella | 1/5 (20.0%) | 0/7 (0.0%) | 1/6 (16.7%) | 2/7 (28.6%) | 4/25 (16.0%) |
| Methicillin-Sensitive Staphylococcus aureus (MSSA) | 0/5 (0.0%) | 2/7 (28.6%) | 0/6 (0.0%) | 0/7 (0.0%) | 2/25 (8.0%) |
| Methicillin Resistant Staphylococcus aureus (MRSA) | 0/5 (0.0%) | 2/7 (28.6%) | 0/6 (0.0%) | 0/7 (0.0%) | 2/25 (8.0%) |
| Pseudomonas species | 1/5 (20.0%) | 0/7 (0.0%) | 0/6 (0.0%) | 1/7 (14.3%) | 2/25 (8.0%) |
| Legionella | 1/5 (20.0%) | 0/7 (0.0%) | 0/6 (0.0%) | 1/7 (14.3%) | 2/25 (8.0%) |
| Other Streptococci | 0/5 (0.0%) | 0/7 (0.0%) | 1/6 (16.7%) | 0/7 (0.0%) | 1/25 (4.0%) |
| Haemophilus | 0/5 (0.0%) | 0/7 (0.0%) | 1/6 (16.7%) | 0/7 (0.0%) | 1/25 (4.0%) |
| Enterobacter | 0/5 (0.0%) | 0/7 (0.0%) | 1/6 (16.7%) | 0/7 (0.0%) | 1/25 (4.0%) |
| Other | 2/ 5 (40.0%) | 1/ 7 (14.3%) | 1/ 6 (16.7%) | 2/ 7 (28.6%) | 6/ 25 (24.0%) |
|  | | | | | |
|  | | | | | |
| **Site - Intra abdominal** |  |  |  |  |  |
| Enterococcus species | 0/1 (0.0%) | 1/5 (20.0%) | 0/2 (0.0%) | 1/3 (33.3%) | 2/11 (18.2%) |
| Escherichia coli (E. coli) | 0/1 (0.0%) | 1/5 (20.0%) | 0/2 (0.0%) | 1/3 (33.3%) | 2/11 (18.2%) |
| Mixed | 0/1 (0.0%) | 0/5 (0.0%) | 1/2 (50.0%) | 1/3 (33.3%) | 2/11 (18.2%) |
| Other Streptococci | 0/1 (0.0%) | 1/5 (20.0%) | 0/2 (0.0%) | 0/3 (0.0%) | 1/11 (9.1%) |
| Other gram positives | 1/1 (100.0%) | 0/5 (0.0%) | 0/2 (0.0%) | 0/3 (0.0%) | 1/11 (9.1%) |
| Klebsiella | 0/1 (0.0%) | 0/5 (0.0%) | 1/2 (50.0%) | 0/3 (0.0%) | 1/11 (9.1%) |
| Enterobacter | 0/1 (0.0%) | 1/5 (20.0%) | 0/2 (0.0%) | 0/3 (0.0%) | 1/11 (9.1%) |
| Serratia | 0/1 (0.0%) | 1/5 (20.0%) | 0/2 (0.0%) | 0/3 (0.0%) | 1/11 (9.1%) |
|  | | | | | |
|  | | | | | |
| **Site - Blood** |  |  |  |  |  |
| Methicillin-Sensitive Staphylococcus aureus (MSSA) | 5/9 (55.6%) | 0/2 (0.0%) | 0/4 (0.0%) | 0/6 (0.0%) | 5/21 (23.8%) |
| Other Streptococci | 0/9 (0.0%) | 0/2 (0.0%) | 1/4 (25.0%) | 2/6 (33.3%) | 3/21 (14.3%) |
| Coagulase negative staphylococcus | 0/9 (0.0%) | 0/2 (0.0%) | 0/4 (0.0%) | 2/6 (33.3%) | 2/21 (9.5%) |
| Other gram positives | 0/9 (0.0%) | 1/2 (50.0%) | 1/4 (25.0%) | 0/6 (0.0%) | 2/21 (9.5%) |
| Klebsiella | 1/9 (11.1%) | 0/2 (0.0%) | 0/4 (0.0%) | 1/6 (16.7%) | 2/21 (9.5%) |
| Escherichia coli (E. coli) | 0/9 (0.0%) | 0/2 (0.0%) | 2/4 (50.0%) | 0/6 (0.0%) | 2/21 (9.5%) |
| Streptococcus pneumoniae (or Pneumococcus) | 0/9 (0.0%) | 0/2 (0.0%) | 0/4 (0.0%) | 1/6 (16.7%) | 1/21 (4.8%) |
| Enterococcus species | 0/9 (0.0%) | 1/2 (50.0%) | 0/4 (0.0%) | 0/6 (0.0%) | 1/21 (4.8%) |
| Enterobacter | 1/9 (11.1%) | 0/2 (0.0%) | 0/4 (0.0%) | 0/6 (0.0%) | 1/21 (4.8%) |
| Other | 2/ 9 (22.2%) | 0/ 2 ( 0.0%) | 0/ 4 ( 0.0%) | 0/ 6 ( 0.0%) | 2/ 21 ( 9.5%) |
|  | | | | | |
|  | | | | | |
| **Site - Skin** |  |  |  |  |  |
| Other Streptococci | 3/4 (75.0%) | 1/5 (20.0%) | 0/2 (0.0%) | 0/1 (0.0%) | 4/12 (33.3%) |
| Other gram positives | 0/4 (0.0%) | 1/5 (20.0%) | 1/2 (50.0%) | 0/1 (0.0%) | 2/12 (16.7%) |
| Other | 0/4 (0.0%) | 1/5 (20.0%) | 0/2 (0.0%) | 1/1 (100.0%) | 2/12 (16.7%) |
| Methicillin Resistant Staphylococcus aureus (MRSA) | 0/4 (0.0%) | 1/5 (20.0%) | 0/2 (0.0%) | 0/1 (0.0%) | 1/12 (8.3%) |
| Enterobacter | 1/4 (25.0%) | 0/5 (0.0%) | 0/2 (0.0%) | 0/1 (0.0%) | 1/12 (8.3%) |
| Other gram negatives | 0/4 (0.0%) | 0/5 (0.0%) | 1/2 (50.0%) | 0/1 (0.0%) | 1/12 (8.3%) |
| Mixed | 0/4 (0.0%) | 1/5 (20.0%) | 0/2 (0.0%) | 0/1 (0.0%) | 1/12 (8.3%) |
|  | | | | | |
|  | | | | | |
| **Site - Urinary** |  |  |  |  |  |
| Escherichia coli (E. coli) | 1/3 (33.3%) | 1/3 (33.3%) | 2/6 (33.3%) | 4/6 (66.7%) | 8/18 (44.4%) |
| Enterococcus species | 1/3 (33.3%) | 0/3 (0.0%) | 1/6 (16.7%) | 0/6 (0.0%) | 2/18 (11.1%) |
| Klebsiella | 0/3 (0.0%) | 1/3 (33.3%) | 1/6 (16.7%) | 0/6 (0.0%) | 2/18 (11.1%) |
| Other gram negatives | 0/3 (0.0%) | 0/3 (0.0%) | 2/6 (33.3%) | 0/6 (0.0%) | 2/18 (11.1%) |
| Other gram positives | 0/3 (0.0%) | 0/3 (0.0%) | 0/6 (0.0%) | 1/6 (16.7%) | 1/18 (5.6%) |
| Enterobacter | 0/3 (0.0%) | 1/3 (33.3%) | 0/6 (0.0%) | 0/6 (0.0%) | 1/18 (5.6%) |
| Serratia | 0/3 (0.0%) | 0/3 (0.0%) | 0/6 (0.0%) | 1/6 (16.7%) | 1/18 (5.6%) |
| Candida | 1/3 (33.3%) | 0/3 (0.0%) | 0/6 (0.0%) | 0/6 (0.0%) | 1/18 (5.6%) |
|  | | | | | |
|  | | | | | |
| **Site - Gut** |  |  |  |  |  |
| Enterococcus species | 1/2 (50.0%) | 0/1 (0.0%) | 0/0 (0.0%) | 0/1 (0.0%) | 1/4 (25.0%) |
| Pseudomonas species | 1/2 (50.0%) | 0/1(0.0%) | 0/0 (0.0%) | 0/1 (0.0%) | 1/4 (25.0%) |
| Escherichia coli (E. coli) | 0/2 (0.0%) | 0/1 (0.0%) | 0/0 (0.0%) | 1/1 (100.0%) | 1/4 (25.0%) |
| Bacteroides | 0/2 (0.0%) | 1/1 (100.0%) | 0/0 (0.0%) | 0/1 (0.0%) | 1/4 (25.0%) |
|  | | | | | |
|  | | | | | |
| **Site - Endocarditis** |  |  |  |  |  |
| Methicillin-Sensitive Staphylococcus aureus (MSSA) | 0/0 (0.0%) | 1/1 (100.0%) | 0/0 (0.0%) | 0/0 (0.0%) | 1/1 (100.0%) |
|  | | | | | |
|  | | | | | |
| **Site - Other** |  |  |  |  |  |
| Other Streptococci | 0/0 (0.0%) | 3/3 (100.0%) | 0/2 (0.0%) | 0/0 (0.0%) | 3/5 (60.0%) |
| Methicillin-Sensitive Staphylococcus aureus (MSSA) | 0/0 (0.0%) | 0/3 (0.0%) | 1/2 (50.0%) | 0/0 (0.0%) | 1/5 (20.0%) |
| Other gram positives | 0/0 (0.0%) | 0/3 (0.0%) | 1/2 (50.0%) | 0/0 (0.0%) | 1/5 (20.0%) |
|  | | | | | |
|  | | | | | |
| **Overall Bacteraemia rate** | 16/38 (42.1%) | 14/42 (33.3%) | 10/36 (27.8%) | 12/37 (32.4%) | 52/153 (34.0%) |
|  | | | | | |

**Table S8 - Compliance to study treatment**

|  | **Fludrocortisone __________________________________________________________** | | |
| --- | --- | --- | --- |
|  | **50 mcg (N=42)** | **100 mcg (N=36)** | **200 mcg (N=37)** |
|  | | | |
|  |  |  |  |
| **Stat dose of study fludrocortisone** |  |  |  |
| Stat dose given within 1 hour of randomisation | 31 (73.8%) | 25 (69.4%) | 23 (62.2%) |
| Stat dose given outside 1 hour of randomisation | 10 (23.8%) | 9 (25.0%) | 13 (35.1%) |
| No stat dose given | 1 (2.4%) | 2 (5.6%) | 1 (2.7%) |
|  | | | |
|  | | | |
|  |  |  |  |
| **Cumulative dose of study fludrocortisone received per patient study day (mcg)** |  |  |  |
| Median (Q1; Q3) | 50.0 (50.0; 50.0) | 100.0 (50.0; 100.0) | 200.0 (100.0; 200.0) |
| min max | 0 100 | 50 150 | 50 200 |
|  | | | |
|  | | | |
| **Overall compliance to study drug (%)** | 98.4 ± 12.73 | 94.0 ± 16.30 | 93.9 ± 18.16 |
|  | | | |
|  | | | |
| **Missed doses of study fludrocortisone** | 1 (0.1%) | 9 (1.4%) | 20 (2.6%) |
|  | | | |
|  | | | |
| **Missed dose due to no enteral access^1^** | 4 (0.6%) | 12 (1.9%) | 3 (0.4%) |
|  | | | |
|  | | | |
|  | | | |

**Table S9 - Adverse events**

|  | **Fludrocortisone __________________________________________________** | | | |  |
| --- | --- | --- | --- | --- | --- |
|  | **0 mcg (N=38) Event n (%)** | **50 mcg (N=42) Event n (%)** | **100 mcg (N=36) Event n (%)** | **200 mcg (N=37) Event n (%)** | **Total (N=153) Event n (%)** |
| **Any adverse event** | 0 (0.0%) | 1 (2.4%) | 0 (0.0%) | 1 (2.6%) | 2 (1.3%) |
|  | | | | | |
| **Any serious adverse event** | 0 (0.0%) | 0 (0.0%) | 0 (0.0%) | 0 (0.0%) | 0 (0.0%) |
|  | | | | | |
|  |  |  |  |  |  |
| **Event related to the study drug** |  |  |  |  |  |
| Possibly related | 0 (0.0%) | 1 (2.0%) | 0 (0.0%) | 0 (0.0%) | 1 (0.6%) |
| Probably related | 0 (0.0%) | 0 (0.0%) | 0 (0.0%) | 1 (2.6%) | 1 (0.6%) |
| Definitely related | 0 (0.0%) | 0 (0.0%) | 0 (0.0%) | 0 (0.0%) | 0 (0.0%) |
|  | | | | | |
|  |  |  |  |  |  |
| **Outcome of the event** |  |  |  |  |  |
| Unknown | 0 (0.0%) | 0 (0.0%) | 0 (0.0%) | 0 (0.0%) | 0 (0.0%) |
| Resolved spontaneously | 0 (0.0%) | 0 (0.0%) | 0 (0.0%) | 1 (2.6%) | 1 (0.6%) |
| Resolved with treatment | 0 (0.0%) | 1 (2.0%) | 0 (0.0%) | 0 (0.0%) | 1 (0.6%) |
| Death | 0 (0.0%) | 0 (0.0%) | 0 (0.0%) | 0 (0.0%) | 0 (0.0%) |
| No resolution (ongoing) | 0 (0.0%) | 0 (0.0%) | 0 (0.0%) | 0 (0.0%) | 0 (0.0%) |
| Resolved with sequelae | 0 (0.0%) | 0 (0.0%) | 0 (0.0%) | 0 (0.0%) | 0 (0.0%) |
|  | | | | | |

One patient in the FC 200 µg group developed metabolic alkalosis and one patient in the FC 50µg group developed hypernatraemia

**Table S10 – Daily laboratory and organ failure data**

|  | **Fludrocortisone** | | |
| --- | --- | --- | --- |
| **Outcome** | **50 mcg vs. Control** | **100 mcg vs. Control** | **200 mcg vs. Control** |
|  | | | |
| **Lowest serum sodium (mmol/L)^1.MD*^** | -0.28 (-1.96; 1.39) p=(0.7404) | 0.28 (-1.45; 2.02) p=(0.7488) | -0.86 (-2.56; 0.85) p=(0.3230) |
| **Highest serum sodium (mmol/L)^1.MD*^** | -1.04 (-2.76; 0.67) p=(0.2321) | -0.86 (-2.63; 0.91) p=(0.3381) | -0.82 (-2.57; 0.93) p=(0.3598) |
| **Lowest serum potassium (mmol/L)^1.MD*^** | -0.06 (-0.16; 0.04) p=(0.2740) | -0.09 (-0.20; 0.02) p=(0.0944) | -0.01 (-0.12; 0.09) p=(0.8075) |
| **Highest serum potassium (mmol/L)^1.MD*^** | -0.01 (-0.13; 0.10) p=(0.7966) | -0.00 (-0.12; 0.12) p=(0.9965) | 0.02 (-0.09; 0.14) p=(0.7089) |
| **Highest serum creatinine (umol/L)^1.MD*^** | 5.58 (-28.8;39.97) p=(0.7501) | -14.2 (-50.1;21.71) p=(0.4378) | -17.5 (-53.0;18.01) p=(0.3337) |
| **Highest bilirubin (umol/L)^1.MD*^** | 8.29 (-10.7;27.27) p=(0.3911) | 5.62 (-14.1;25.37) p=(0.5768) | 5.62 (-13.9;25.16) p=(0.5727) |
| **Lowest platelet count (x10^9^/L)^1.MD*^** | 19.28 (-20.5;59.05) p=(0.3417) | 20.68 (-20.4;61.79) p=(0.3235) | 12.02 (-28.9;52.93) p=(0.5642) |
| **Last arterial lactate (mmol/L)^1.MD*^** | 0.14 (-0.29; 0.58) p=(0.5182) | 0.24 (-0.22; 0.70) p=(0.3004) | -0.02 (-0.48; 0.43) p=(0.9157) |
| **Urine output (ml)^1.MD^** | -172 ( -649;304.3) p=(0.4778) | -189 ( -687;308.3) p=(0.4552) | -217 ( -709;275.7) p=(0.3879) |
| **Fluid balance (ml)^1.MD^** | -126 ( -536;284.0) p=(0.5471) | 241.6 ( -190;673.1) p=(0.2722) | -76.0 ( -500;348.5) p=(0.7254) |
| **Last heart rate^1.MD*^** | 0.37 (-4.80; 5.55) p=(0.8870) | 1.27 (-4.17; 6.71) p=(0.6465) | 5.47 ( 0.09;10.85) p=(0.0463) |
| **Lowest MAP (mmHg)^1.MD*^** | -1.17 (-3.91; 1.57) p=(0.4018) | -0.40 (-3.25; 2.45) p=(0.7831) | 0.27 (-2.54; 3.08) p=(0.8506) |
| **Lowest ventilated PaO2 / FIO2 ratio^1.MD*^** | -1.85 (-29.0;25.35) p=(0.8939) | -4.36 (-33.2;24.42) p=(0.7660) | -13.8 (-42.3;14.68) p=(0.3415) |
| **Glasgow coma scale (unsedated)^1.MD*^** | 0.02 (-1.39; 1.43) p=(0.9786) | 0.69 (-0.78; 2.17) p=(0.3551) | -0.19 (-1.67; 1.29) p=(0.8053) |
| **Hyponatremia (Lowest serum Na<135 mmol/L)^2.OR*^** | 0.98 ( 0.29; 3.31) p=(0.9771) | 0.64 ( 0.17; 2.39) p=(0.5019) | 1.40 ( 0.40; 4.92) p=(0.6039) |
| **Hypernatremia (Highest serum Na>150 mmol/L)^2.OR*^** | 0.21 ( 0.02; 1.76) p=(0.1498) | 0.40 ( 0.04; 3.74) p=(0.4252) | 0.54 ( 0.06; 4.75) p=(0.5786) |
| **Hypokalemia (Lowest serum K<3.5 mmol/L)^2.OR*^** | 1.48 ( 0.71; 3.07) p=(0.2904) | 2.09 ( 0.97; 4.52) p=(0.0614) | 1.39 ( 0.65; 2.94) p=(0.3924) |
| **Hyperkalemia (Highest serum K>5.0 mmol/L)^2.OR*^** | 1.31 ( 0.43; 3.99) p=(0.6293) | 0.98 ( 0.29; 3.27) p=(0.9692) | 1.56 ( 0.49; 4.94) p=(0.4528) |

Figure S1 - Study flowchart

Screened patients n=422

**Reasons for exclusion (n=267)**

Met all inclusion criteria > 24 hrs – 41

Patients on long term steroids – 68

Death deemed inevitable during current admission or within 90 days – 51

Unable to receive enteral medication - 37

Eligible but not randomized – 59

Miscellaneous - 11

Fludrocortisone 100mcg n=36

Fludrocortisone 200mcg n=37

Fludrocortisone 50mcg n=42

ITT population n=153

Randomised patients n=155

Consent declined n=2

Fludrocortisone 0mcg

n=38

**Day 28:**

Alive n=35

- Alive in ICU n=5
- Alive & discharged from hospital n=18

Deaths n=7

Lost to follow-up n=0

**Day 28:**

Alive n=29

- Alive in ICU n=1
- Alive & discharged from hospital n=18

Deaths n=9

Lost to follow-up n=0

**Day 28:**

Alive n=33

- Alive in ICU n=4
- Alive & discharged from hospital n=22

Deaths n=4

Lost to follow-up n=0

**Day 28:**

Alive n=32

- Alive in ICU n=3
- Alive & discharged from hospital n=16

Deaths n=4

Lost to follow-up n=0

**Figure S2 – Mean plots of the lowest MAP, heart rate and Vasoactive Inotropic Score over time.**

**Figure S2A**

**
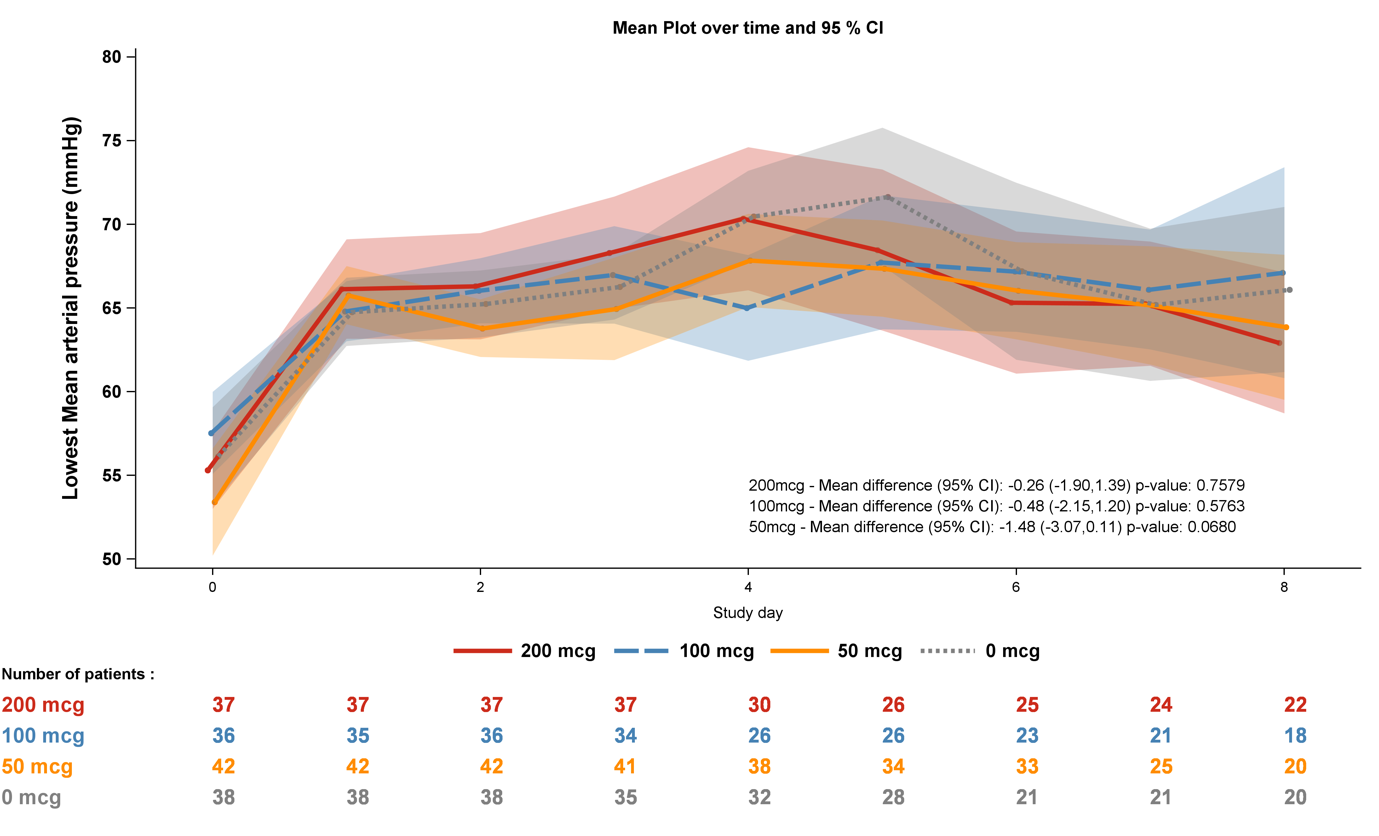
**

**Figure S2B**

**
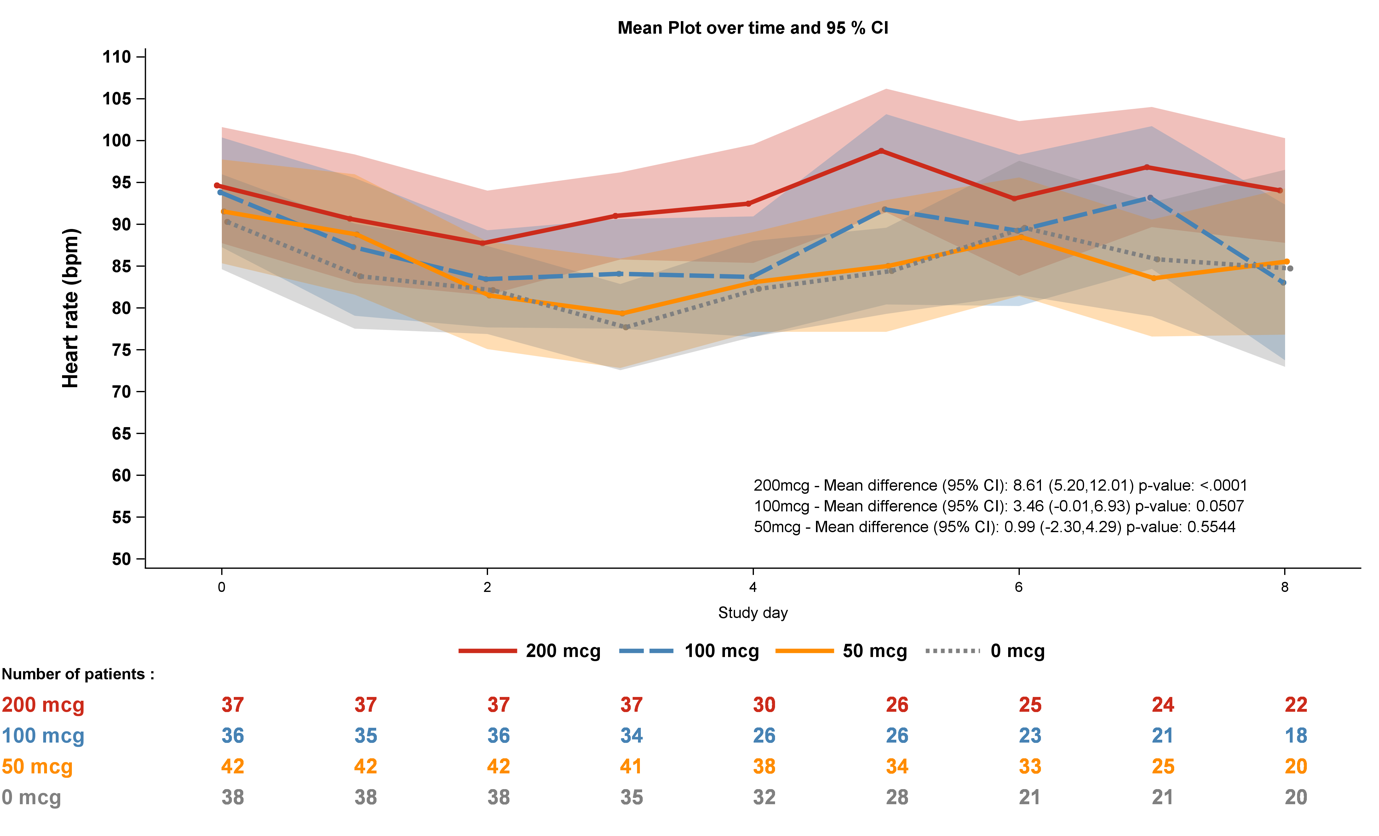
**

**Figure S2C**

**
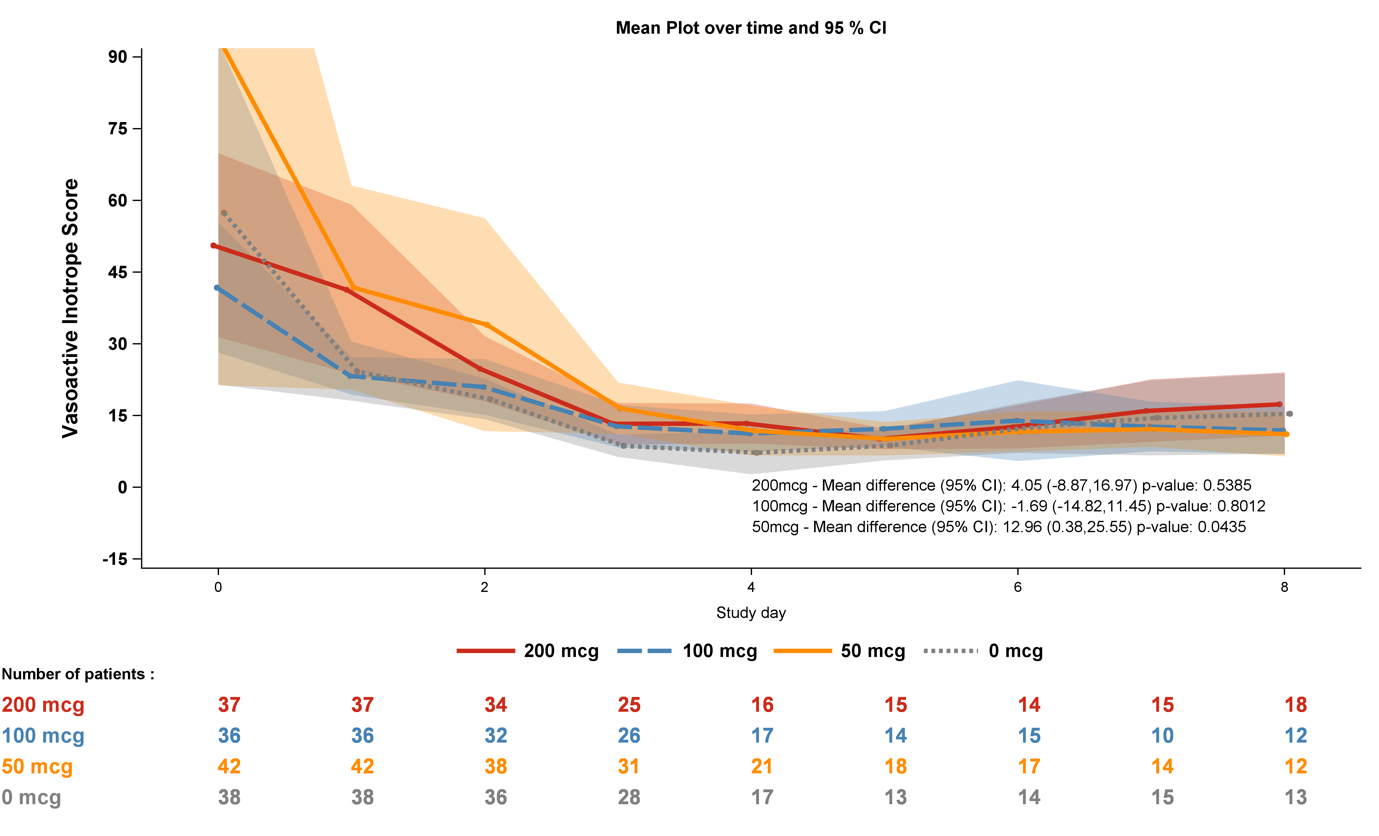
**

**Figure S3 – Cumulative incidence function of time to cessation of mechanical ventilation, ICU discharge and hospital discharge**

**Figure S3A**

**
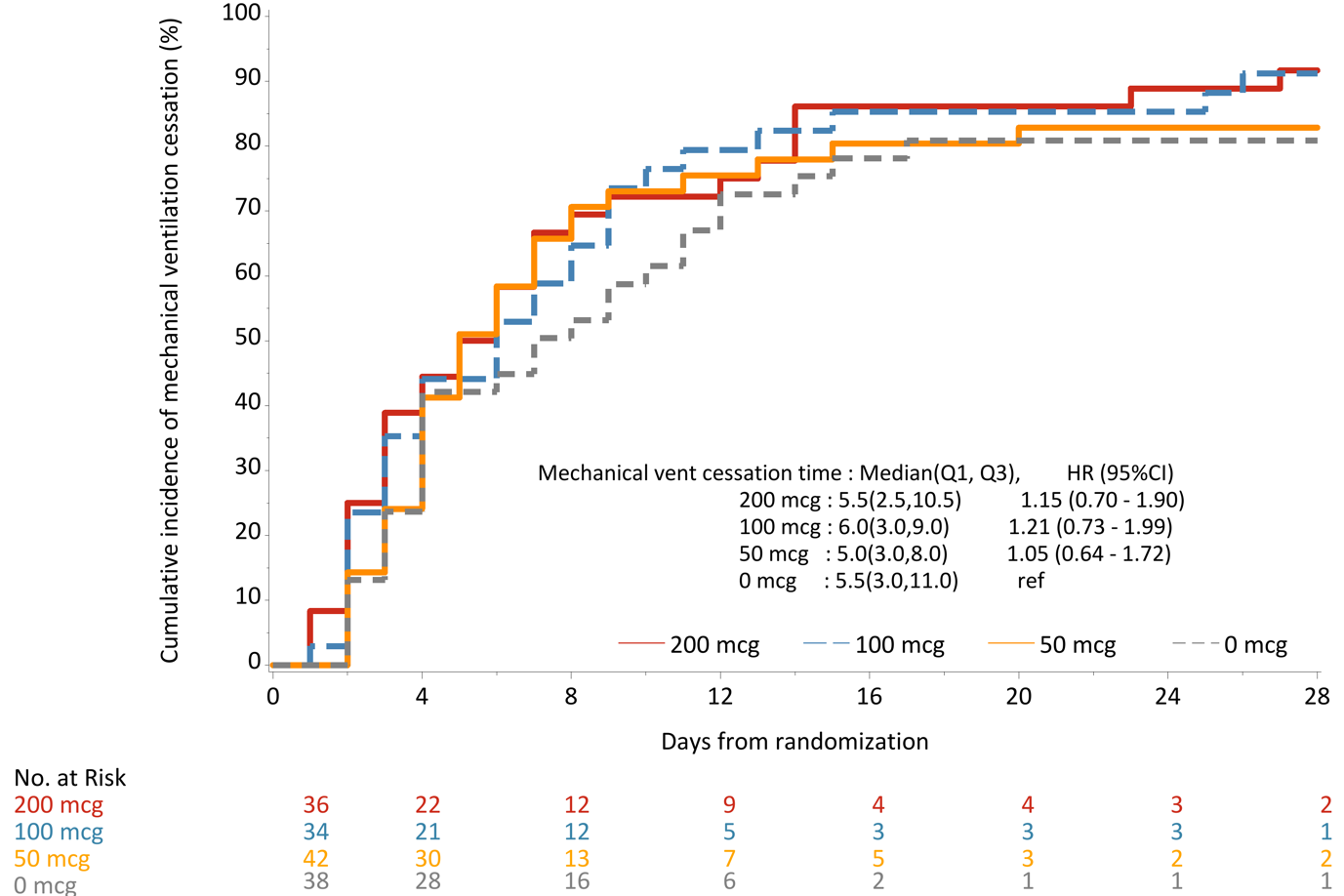
**

**Figure S3B**

**
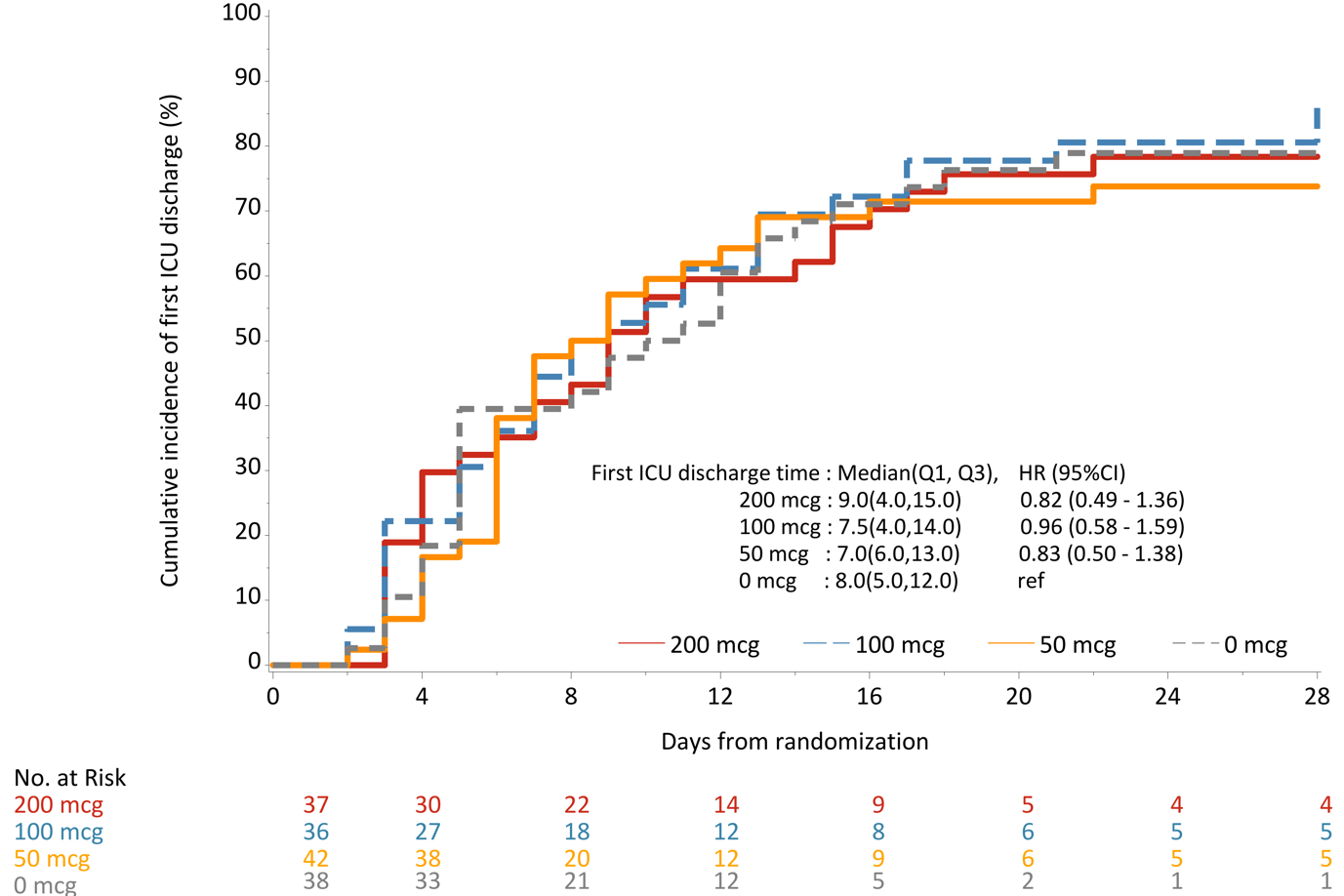
**

**Figure S3C**

**
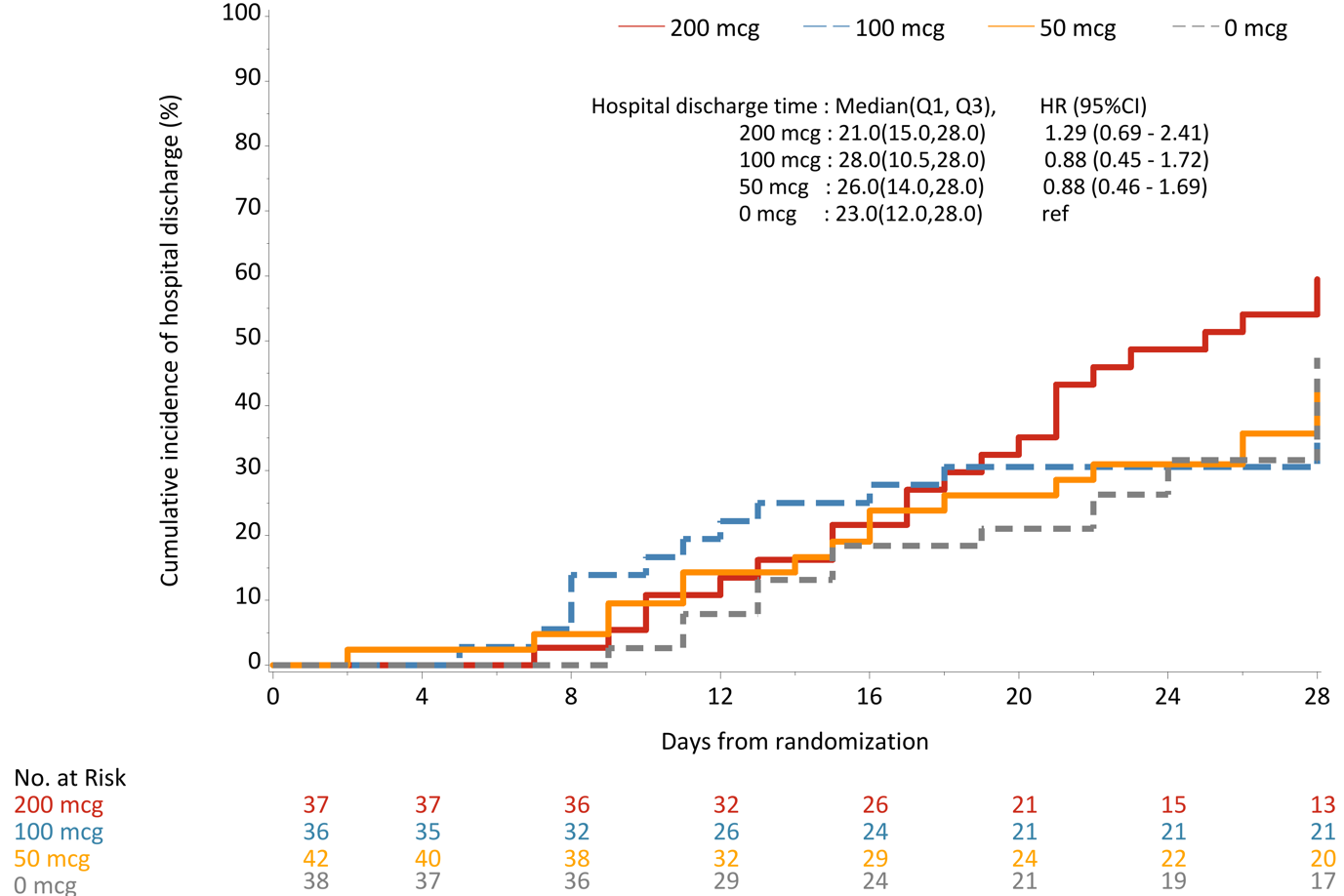
**

**Figure S4 – Plasma fludrocortisone levels at time 0 and 3 hours in each of the 3 dosing groups.**

Figure S4: Observed fludrocortisone (FC) plasma concentrations quantified for all subjects in this study at 0 (closed symbols) and 3 h (open symbols) for 50 (▲△), 100 (■☐) and 200 (⚫〇) mcg on oral/or via gastric tube multiple dosing for up to 5 days, after pre-dosing superimposed on the predicted FC plasma concentration - time profiles based on the intravenous and oral data for a similar patient population collected over 24 hours by Vogt et al.^1^ and the oral multiple dosing pharmacokinetic equation 445 in Gibaldi and Perrier^2^

1. Vogt VW, Fischer I, Ebenroth S, Appel S, Knedel M, Lücker P, et al. Zur Pharmakokinetik von 9α-Fluorhydrocortison. Arzneimittel-Forschung. 1971 Aug;21(8):S1133-43.

2. Gibaldi, M and Perrier, D; “Pharmacokinetics” vol.1 Marcel Dekker, NY (1975) chap.3: Multiple Dosing.

**Figure S5: A plot of VIS vs plasma fludrocortisone concentrations at time 0 and 3 hrs.**


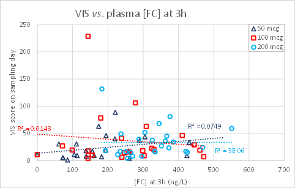


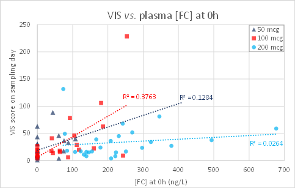


Observed fludrocortisone (FC) plasma concentrations quantified for all subjects in this study at 0 (closed symbols) and 3 h (open symbols) for 50 (▲△), 100 (■☐) and 200 (⚫〇) mcg on the X-axis and VIS scores on the y-axis.

1. Baymeeva NV, Platova AI, Miroshnichenko II, Belovolov AYu, Gladkikh VD and Tatarinov AM (2021) *Pharmaceutical Chemistry Journal*, 55(5):510–515, doi:[10.1007/s11094-021-02453-6](https://doi.org/10.1007/s11094-021-02453-6). [↑](#footnote-ref-1)
